# TFEB inhibition induces melanoma shut-down by blocking the cell cycle and rewiring metabolism

**DOI:** 10.1038/s41419-023-05828-7

**Published:** 2023-05-09

**Authors:** C. Ariano, F. Costanza, M. Akman, C. Riganti, D. Corà, E. Casanova, E. Astanina, V. Comunanza, F. Bussolino, G. Doronzo

**Affiliations:** 1 Department of Oncology, University of Torino, Torino, Italy; 2 Candiolo Cancer Institute- FPO-IRCCS, Candiolo, Italy; 3 Department of Translational Medicine, Piemonte Orientale University, Novara, Italy; 4 Center for Translational Research on Autoimmune and Allergic Diseases - CAAD, Novara, Italy

**Keywords:** Cancer metabolism, Cell-cycle exit

## Abstract

Melanomas are characterised by accelerated cell proliferation and metabolic reprogramming resulting from the contemporary dysregulation of the MAPK pathway, glycolysis and the tricarboxylic acid (TCA) cycle. Here, we suggest that the oncogenic transcription factor EB (TFEB), a key regulator of lysosomal biogenesis and function, controls melanoma tumour growth through a transcriptional programme targeting ERK1/2 activity and glucose, glutamine and cholesterol metabolism. Mechanistically, TFEB binds and negatively regulates the promoter of DUSP-1, which dephosphorylates ERK1/2. In melanoma cells, TFEB silencing correlates with ERK1/2 dephosphorylation at the activation-related p-Thr185 and p-Tyr187 residues. The decreased ERK1/2 activity synergises with TFEB control of CDK4 expression, resulting in cell proliferation blockade. Simultaneously, TFEB rewires metabolism, influencing glycolysis, glucose and glutamine uptake, and cholesterol synthesis. In TFEB-silenced melanoma cells, cholesterol synthesis is impaired, and the uptake of glucose and glutamine is inhibited, leading to a reduction in glycolysis, glutaminolysis and oxidative phosphorylation. Moreover, the reduction in TFEB level induces reverses TCA cycle, leading to fatty acid production. A syngeneic BRAFV600E melanoma model recapitulated the in vitro study results, showing that TFEB silencing sustains the reduction in tumour growth, increase in DUSP-1 level and inhibition of ERK1/2 action, suggesting a pivotal role for TFEB in maintaining proliferative melanoma cell behaviour and the operational metabolic pathways necessary for meeting the high energy demands of melanoma cells.

## Introduction

Melanoma is the cancer most frequently diagnosed in young adults [1] with the risk of metastatic spread that is high, although most patients show localised lesions at the time of diagnosis and are successfully treated by surgery [1].

In skin melanomas, mitogen-activated protein kinase (MAPK) signalling is often switched on by activating mutations in genes regulating the pathway [2, 3]. The most common melanoma genomic subtype carries *BRAF* mutations (52%), primarily V600E. Mutations targeting *NRAS* and *NF1* also characterise a large number of cases and affect the MAPK pathway. Other melanomas are categorised as the triple wild-type (WT) subtype, which is characterised mainly by mutations in *KIT*, another factor of the MAPK pathway [3].

Among transcription factors, microphthalmia-associated transcription factor (MITF), a member of the MiT family of bHLH-leucine zipper transcription factors, plays an important role in melanoma onset and progression [4–7].

The MAPK pathway is a kinase cascade downstream of BRAF and consists of mitogen-activated protein kinase kinase (MEK) 1/2, which phosphorylates extracellular signal-regulated kinase (ERK) 1/2 [8, 9]. The ERK1/2 kinase activates many substrates, leading to a broad range of molecular functions involved in cell proliferation, metabolism, survival, growth, migration and differentiation [9, 10].

In melanoma cells, as well as in other cancer cells, the MAPK pathway is the link between uncontrolled cell proliferation and the metabolic rewiring required to sustain the high demands of anabolic processes [11, 12]. ERK1/2 signalling directs cell proliferation and, in particular, the G1/S-phase transition in the cell cycle by activating a complex signalling cascade supported by different transcription factors that modulate the expression of early and secondary genes, including Cyclin D1 (*Ccnd1*) [9, 10, 13, 14]. By interacting with cyclin-dependent kinase (CDK) 4/6, Cyclin D1, inhibits the retinoblastoma (Rb) protein, leading to E2 transcription factor migration into the nucleus, where it activates the transcription of genes involved in DNA replication [13].

The constitutive activation of the BRAF/MAPK pathway [11, 12, 15–18] supports the transcription of glucose transporters (GLUTs) and the synthesis and activation of glycolytic enzymes [11, 12, 15–18]. An increased aerobic glycolysis rate corresponds to reduced entry of carbon into TCA cycle, which is initiated by glutamine consumption to drive the biosynthesis of metabolites needed for reductive carboxylation and anaplerotic reactions [19–21].

Inhibition of BRAFV600E activity reduces the glycolysis and glucose transport rates [18, 22, 23], and these outcomes parallel a reduction MAPK pathway activation [18]. Furthermore, ERK1/2 regulates the behaviour of the M2 isoform of pyruvate kinase (PK) [24–27] the most abundant glycolytic enzyme in proliferating cells, including tumour cells [24–27]. PKM2 is involved in the conversion of phosphoenolpyruvate (PEP) to pyruvate [24–27]. ERK1/2 phosphorylates PKM2, which is transformed from a highly active tetramer to a dimer with low enzymatic activity, functioning as a gene transcription regulator after entering the nucleus, where it phosphorylates proteins, including histones [27].

In addition to MITF [7], transcription factor EB (TFEB), another member of the MiT transcription factor family, has been suggested to play a role in melanoma onset and progression [4–6, 28–31].

TFEB coordinates a transcriptional programme that controls the main degradative pathways to promote intracellular clearance by enhancing lysosome activity and autophagic flux [28–31]. In melanoma cells, MITF and TFEB participate in cross-regulatory circuits, in which MITF enhances the expression of TFEB, while TFEB inhibits MITF expression [32]. Furthermore, in BRAF-mutant melanoma cells, the increased activity of the MAPK pathway results in the inhibition of the genetic programme regulated by TFEB, leading to tumour progression and chemoresistance to BRAF inhibitors [33].

Recent findings have demonstrated new implications of TFEB in cell life including cell proliferation and metabolism, immunity, angiogenesis, inflammation and drug resistance [28–31, 34–52]. In endothelial and HeLa cells, loss-of-function strategies have led to direct TFEB-mediated activation of the CDK4/CyclinD1/Rb protein pathway and the inhibition of cell proliferation [39, 40]. Moreover, in endothelial cells, TFEB is involved in the regulation of cholesterol synthesis and in cholesterol distribution in the plasma membrane [35, 39].

In this study, we demonstrated that TFEB exerts a relevant impact on the behaviour of BRAF-mutated melanomas. The results show combined TFEB direct or indirect transcriptional effects on the MAPK pathway and genes involved in cell cycle regulation and metabolism. TFEB is a repressor of dual-specific phosphatase-1 (DUSP-1), which is a key negative regulator of MAPK in mammalian cells, including melanoma cells [53–56]. Silencing of *Tfeb* under physiological conditions led to an increase in DUSP-1 expression and dephosphorylation of ERK1/2. In parallel, TFEB controlled the cell cycle and cell proliferation directly by regulating *Cdk4* and indirectly via ERK-mediated modulation of *Ccnd1* expression. The metabolic effects of TFEB were found to be multifaceted and encompass aerobic and anaerobic ATP production and cholesterol synthesis. In the absence of TFEB, cholesterol synthesis was impaired, leading to altered uptake rates of glucose and glutamine, probably by changing membrane fluidity. This effect profoundly influenced glycolysis, glutaminolysis, the TCA cycle and mitochondrial ATP production.

Interestingly, the most important hallmarks found in vitro were confirmed in syngeneic BRAFV600E melanoma tumours after *Tfeb* silencing, which resulted in a reduction in tumour growth.

## Results

### TFEB deletion leads to MAPK pathway activity inhibition

Experiments were performed with different murine melanoma cell lines carrying WT *Braf* (the YUMM 4.1 cell line) or *Braf*V600E (the D4M, YUMM 3.3, and YUMM 1.7 cell lines) (Supplementary Fig. S1A) [57, 58]. As expected, BRAFV600E-mutated cells showed constitutive phosphorylation of both MEK1/2 and ERK1/2 (Supplementary Fig. S1A), and interestingly, we found that the TFEB protein levels were reduced to different degrees in the YUMM 4.1 to D4M, YUMM 1.7 and YUMM 3.3 cells (Supplementary Fig. S1A).

**Fig. 1:**
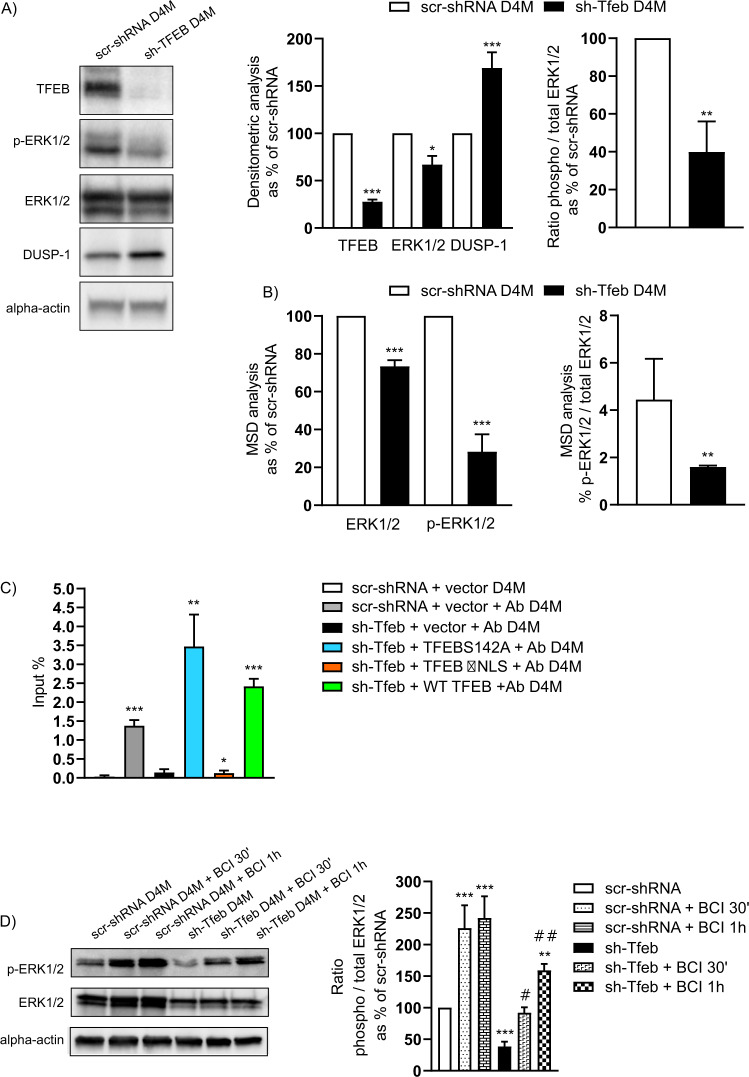
*Tfeb* silencing inhibits the MAPK pathway via DUSP-1 activation. **A** Representative western blots showing TFEB, phosphorylated- and total ERK1/2, and DUSP-1 expression levels in scr-shRNA and sh-Tfeb D4M cells. The bar graphs show the densitometry analysis of TFEB, ERK1/2 and DUSP-1 expression levels and the ratio of the phosphorylated ERK1/2 level to- total ERK1/2 level in sh-Tfeb cells as % of scr-shRNA cells after normalisation to the level of alpha-actin protein (*n* = 3 independent experiments; the means ± SEMs; ***p < 0.0001, **p < 0.001 and *p < 0.01 for sh-Tfeb versus scr-shRNA D4M cells, as determined by Student’s *t* test). **B** ERK1/2 quantity and activity (expressed as the ratio of p-ERK1/2 level to total ERK level) in scr-shRNA and sh-Tfeb D4M cells measured by MSD technology (*n* = 3 independent experiments; the means ± SEMs; ***p < 0.0001 and **p < 0.001 for sh-Tfeb versus scr-shRNA D4M cells, as determined by Student’s *t* test). **C** The bar graph shows the quantification of TFEB binding of the *Dusp-1* promoter in D4M. ChIP was performed using digested chromatin from scr-shRNA/sh-Tfeb +vector, sh-Tfeb D4M after addiction of TFEBS142A or TFEB ∆NLS or WT TFEB incubated or not with Ab anti-TFEB (indicated in the bar graph as “+Ab”), followed by qPCR for *Dusp-1*. Bar graph shows the enrichment percentage (*n* = 3 independent experiments; the values are reported as the means ± SEMs; ***p < 0.0001, **p < 0.001 and *p < 0.01 under all conditions versus scr-shRNA + vector D4M cells, as determined by Student’s *t* test). **D** Representative western blots show phosphorylated- and total ERK1/2 expression in scr-shRNA and sh-Tfeb D4M cells treated or not treated with BCI (1 µM, 30’-1 h). The bar graph shows the densitometry results expressed as the ratio of phosphorylated protein level- to total protein level (*n* = 3 independent experiments; the means ± SEMs; ***p < 0.0001 and **p < 0.001 for untreated sh-Tfeb versus scr-shRNA D4M cells, ^##^*p* < 0.001 and ^#^*p* < 0.01 for treated sh-Tfeb versus treated scr-shRNA D4M cells, as determined by Student’s *t* test).

Endogenous *Tfeb* was silenced in the melanoma cells by the infection of specific short hairpin (sh-)RNA lentivirus (sh-Tfeb), and in all experiments, *Tfeb*-silenced cells were compared with control cells carrying scramble sh-RNA (scr-shRNA) (Fig. 1A, Supplementary Fig. S1B–D). To exclude compensatory effects mediated by the other members of the MiT family of transcription factors, the mRNA and protein expression levels of TFE3, TFEC and MITF were evaluated after *Tfeb* silencing, and no changes in these levels compared with the those in the scr-shRNA cells were found (Supplementary Fig. S1E).

We focused our attention on D4M cells, but key experiments were also performed with YUMM 4.1, YUMM 3.3 and YUMM 1.7 cells to exclude the possible influence of cell-specific dependent effects.

We showed that sh-Tfeb cells were characterised by a small reduction in ERK1/2 protein expression (Fig. 1A, B and Supplementary Fig. S1D) that was not sustained by altered transcription (the relative fold change in *Mapk1* and *Mapk3* expression in sh-Tfeb cells compared with the expression in scr-shRNA D4M cells after level normalisation based on the housekeeping gene *Tbp* was 0.90 ± 0.11 and 0.94 ± 0.12, respectively; values are reported as the means ± SEMs; p = ns as determined by Student’s *t* test; *n* = 3 independent experiments). Moreover, immunoblot analysis (Fig. 1A and Supplementary Fig. S1D) and Meso Scale Discovery (MSD) assay (Fig. 1B) showed a profound reduction in ERK1/2 activation, which was measured as the ratio of the abundance of ERK1/2 phosphorylated at residues Thr185 and Tyr187 versus the total ERK1/2 abundance (Fig. 1A, B and Supplementary Fig. S1D) in sh-Tfeb D4M and YUMM cells.

These results suggested the putative involvement of a phosphatase acting on ERK1/2. Accordingly, we analysed the mRNA expression of a general panel of phosphatases and found that the expression of some DUSP molecules was upregulated in sh-Tfeb D4M cells (Supplementary Fig. S2A).

Among these genes, *Tfeb* silencing in D4M cells and all YUMM cells increased the mRNA (Supplementary Fig. S2B) and protein expression levels of DUSP-1 (Fig. 1A and Supplementary Fig. S1D), which has been reported to dephosphorylate ERK1/2 [53–55].

Interestingly, a bioinformatics analysis based on the chromatin immunoprecipitation (ChIP) sequencing (seq) binding dataset obtained from a murine model [59] highlighted the enrichment of TFEB at the *Dusp-1* promoter region (Supplementary Fig. S2C), which was defined as the sequence (−2500; +2500, mouse mm9 genome) extending from the transcription start site (TSS) of the *Dusp1* transcript variant ENSMUST00000025025. The TFEB-binding peak in the ChIP-seq data was situated −328 and +333 bp from the TSS of the selected *Dusp-1* transcript, overlapping the TSS itself. A possible TFEB-binding motif, *GTCACGTGTC*, predicted with the Jaspar database [60] (matrix MA0692.1, profile score threshold 80%) was identified at the -81 position in relation to the TSS.

Therefore, we evaluated the regulation of DUSP-1 in our model by the ChIP (Fig. 1C) assay. Chip analysis performed in scr-shRNA or in sh-TFEB D4M cells after the addition of WT TFEB (WT-TFEB), the constitutively active nuclear TFEB mutant (TFEBS142A) or the inactive cytosolic TFEB mutant (TFEB ΔNLS) [59, 61, 62] confirmed TFEB binding to the *Dusp-1* promoter (Supplementary Fig. S2D and Fig. 1C).

We further investigated *Dusp-1* promoter activity by luciferase reporter assay in D4M cells carrying luciferase reporter vectors harbouring: (i) the full-length *Dusp-1* promoter; (ii) a promoter lacking the putative TFEB-binding site (Del1) located in a region extending from −328 and +333 bp with respect to the TSS of the selected *Dusp-1* transcript, as determined by ChIP-seq analysis [59]; (iii) a promoter lacking the putative TFEB-binding site and a sequence of 100 bp before and after this binding site (Del2) (Supplementary Fig. S3A). *Tfeb* silencing in D4M cells resulted in the upregulation of *Dusp-1* promoter activity (Supplementary Fig. S3A). Otherwise, TFEBS142 A expression halted *Dusp-1* promoter activity (Supplementary Fig. S3A), and this effect was completely blunted by the deletion of TFEB-binding sites (Supplementary Fig. S3A).

To confirm the role played by DUSP-1 in the control of ERK1/2 activation, we treated sh-Tfeb and scr-shRNA D4M cells with a specific allosteric inhibitor of DUSP-1, (E)-2-benzylidene-3-(cyclohexylamino)−2,3-dihydro-1H-inden-1-one (BCI) [63, 64], and we found that the level of p-ERK1/2 expressed in sh-Tfeb cells was similar to level expressed in scr-shRNA cells (Fig. 1D).

### TFEB deletion leads to inhibited cell proliferation

**Fig. 2:**
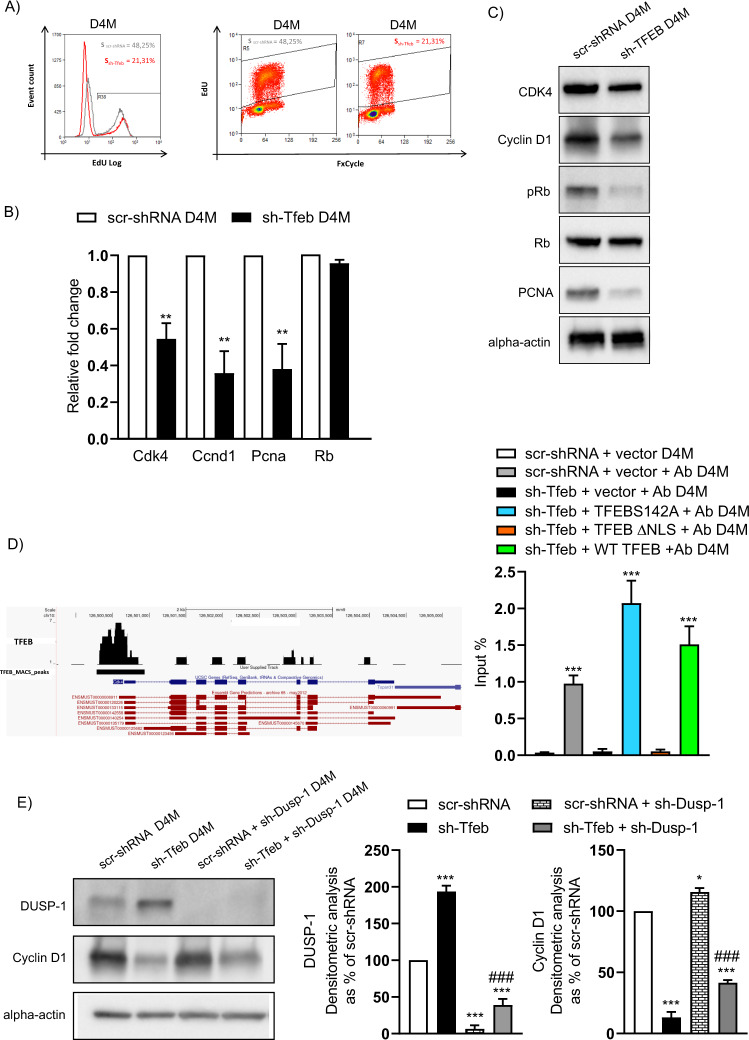
*Tfeb* silencing inhibits melanoma cell proliferation. **A** Flow cytometry evaluation of scr-shRNA and sh-Tfeb D4M cell proliferation determined by measuring cell EdU incorporation during the S phase of the cell cycle. Total DNA content was measured with FxCycle™ Violet Stain. The relative percentage of cells in the S phase is reported. **B** qPCR measurement of *Cdk4, Ccnd1, Pcna and Rb* expression in scr-shRNA and sh-Tfeb D4M cells. The data are expressed as the relative fold change in the sh-Tfeb D4M cells compared with the expression in the scr-shRNA D4M cells after normalisation to the housekeeping gene *Tbp* (*n* = 3 independent experiments, values are reported as the means ± SEMs; **p < 0.001 sh-Tfeb versus scr-shRNA D4M cells by Student’s *t* test). **C** Representative western blots show cellular CDK4, Cyclin D1, Rb, p-Rb, PCNA and alpha-actin expression levels in scr-shRNA and sh-Tfeb D4M cells. **D** Representative snapshot of TFEB binding on *Cdk4* promoter and quantification of TFEB binding of the *Cdk4* promoter in D4M. ChIP was performed using digested chromatin from scr-shRNA/sh-Tfeb+vector, sh-Tfeb D4M after addiction of TFEBS142A or TFEB ∆NLS or WT TFEB incubated or not with Ab anti-TFEB (indicated in the bar graph as “+Ab”), followed by qPCR for *Cdk4*. Bar graph shows the percent enrichment (*n* = 3 independent experiments; values as means ± SEMs; ***p < 0.0001, for all conditions versus scr-shRNA+vector as determined by Student’s *t* test). **E** Representative western blots show the DUSP-1 and Cyclin D1 expression levels in scr-shRNA and sh-Tfeb D4M cells after silencing of *Dusp-1* via sh-RNA lentivirus. The bar graph shows the densitometry results expressed as the ratio of the protein level to the alpha-actin level (*n* = 3 independent experiments; the means ± SEMs; ***p < 0.0001 and *p < 0.01 for all samples versus scr-shRNA D4M cells, ^###^*p* < 0.0001 for sh-Tfeb+sh-Dusp-1 versus sh-Tfeb D4M cells as determined by Student’s *t* test).

*Tfeb* downregulation significantly reduced the cell proliferation rate by inhibiting the G1-S cell cycle transition in D4M and YUMM cells (Fig. 2A and Supplementary Fig. S3B), as previously reported in human endothelial cells [39]. In particular, the number of 5-ethynyl-2′-deoxyuridine (EdU)-positive cells clearly showed a decreased percentage of sh-Tfeb cells moving into the S phase (Fig. 2A and Supplementary Fig. S3B).

In D4M and YUMM sh-Tfeb cells, Cyclin D1 and CDK4 expression was downregulated (Fig. 2B, C and Supplementary Fig. S3C). The analysis of the ChIP-seq binding dataset [59] did not show enrichment of TFEB at the *Ccnd1* promoter region (Supplementary Fig. S3D) but suggested direct binding to the *Cdk4* promoter (Fig. 2D), as previously described in endothelial cells [39]. The *Cdk4* promoter was defined as the sequence (−2500; +2500, mouse mm9 genome) extending from the TSS in the *Cdk4* transcript variant ENSMUST00000120226. A TFEB-binding peak in to ChIP-seq data was located −376 and +278 bp from the TSS of the selected transcript, overlapping the TSS. A possible TFEB-binding motif, *GTCACATGG*, predicted with the Jaspar database [60] (matrix MA0692.1, profile score threshold 80%) was detected at the −14 position in relation to the TSS.

These data were confirmed by ChIP assay with D4M cells (Fig. 2D).

Performing luciferase reporter assays, we further investigated *Cdk4* promoter activity in D4M cells carrying luciferase reporter vectors harbouring: (i) the full-length *Cdk4* promoter; (ii) a promoter lacking the putative TFEB-binding site (Del1), as determined by ChIP-seq analysis, −376 and +278 bps from TSS of selected *Cdk4* transcript [59]; (iii) a promoter lacking the putative TFEB-binding site and a sequence of 100 bp before and after the same binding site (Del2) (Supplementary Fig. S3A). *Tfeb* silencing in D4M cells resulted in the downregulation of *Cdk4* promoter activity (Supplementary Fig. S3A), while TFEBS142A addiction resulted in a significant increase in *Cdk4* promoter activity (Supplementary Fig. S3A). The effect of TFEBS142A was completely blunted by the deletion of TFEB-binding sites (Supplementary Fig. S3A).

Sh-Tfeb D4M cells showed a reduced ratio of pRb/Rb protein (densitometric analysis as % of scr-shRNA D4M: values as means ± SEMs 45 ± 3.2 %; *n* = 3 independent experiments; ***p < 0.0001 for sh-Tfeb versus scr-shRNA cells, as determined by Student’s *t* test) but not the rate of Rb transcription (Fig. 2B) or protein expression (Fig. 2C). As a consequence of the reduced Rb phosphorylation and E2f inactivation, the expression of PCNA, which is an E2f-responsive gene required for DNA synthesis [13], was significantly decreased in D4M and YUMM sh-Tfeb cells (Fig. 2B, C and Supplementary Fig. S3C).

*Dusp-1* silencing in sh-Tfeb D4M cells (Supplementary Fig. S4A, Supplementary Fig. S4B and Fig. 2E) partially rescued Cyclin D1 expression (Fig. 2E), but as expected on the basis of the simultaneous blockade of CDK4 expression, failed to re-establish the G1/S cell cycle transition in the sh-Tfeb cells (Supplementary Fig. S4C).

### TFEB influences melanoma cell glycolysis

**Fig. 3:**
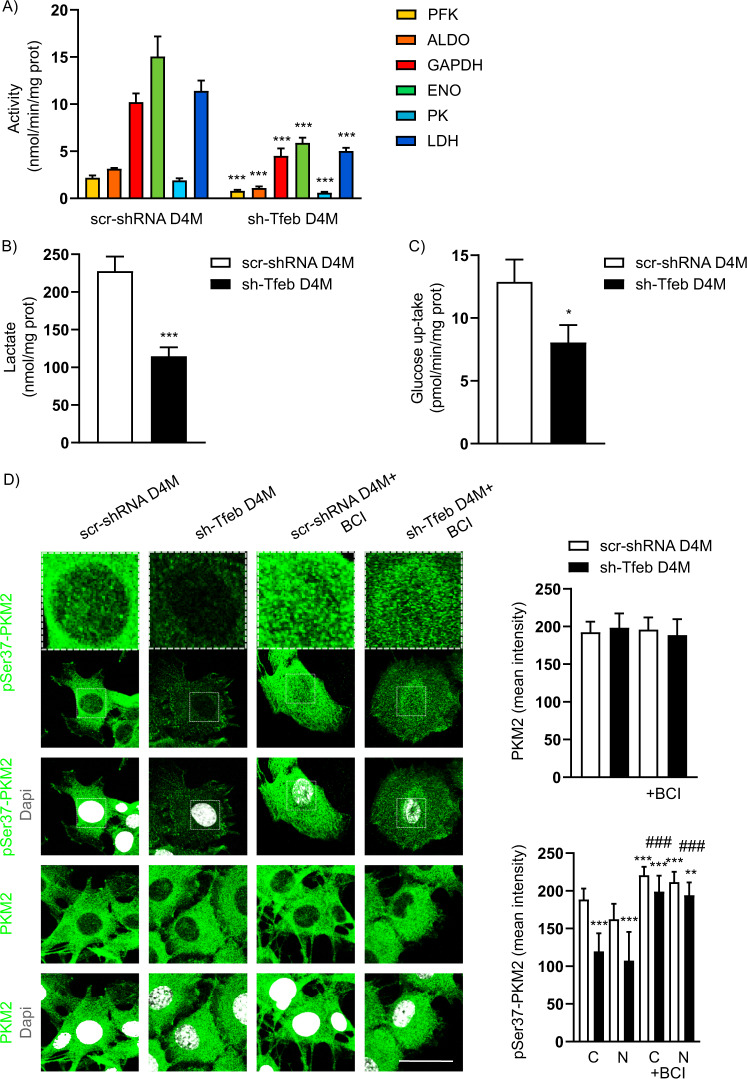
*Tfeb* silencing leads to inhibited glycolysis. **A** The bar graph shows the activity of enzymes involved in glucose metabolism in scr-shRNA and sh-Tfeb D4M cells (the activity of PFK, ALDO, ENO, PK and LDH was measured as nmol NAD^+^/min/mg protein; the activity of GAPDH was measured as nmol NADH/min/mg protein) (*n* = 3 independent experiments; values are reported as the means ± SEMs; ***p < 0.0001 for sh-Tfeb versus scr-shRNA D4M cells, as determined by Student’s *t* test). **B** The bar graphs show the level of lactate in scr-shRNA and sh-Tfeb D4M cells (*n* = 3 independent experiments; values are reported as the means ± SEMs; ***p < 0.0001 for sh-Tfeb versus scr-shRNA D4M cells, as determined by Student’s *t* test). **C** The bar graphs show the quantification of glucose uptake by scr-shRNA and sh-Tfeb D4M cells (*n* = 3 independent experiments; values are reported as the means ± SEMs; *p < 0.01 for sh-Tfeb versus scr-shRNA D4M cells, as determined by Student’s *t* test). **D** Confocal microscopy analysis of pSer37 PKM2 and total PKM2 expression levels in scr-shRNA and sh-Tfeb D4M cells treated with or without BCI (1 µM, 30’-1 h) after incubation with specific antibodies (scale bar: 25 µm). The bar graphs show the mean intensity of the PKM2 signal in whole cells and that of p-PKM2 in the cytosol (C) and nucleus (N) in each cell set (*n* = 15 cells per condition pooled from three different experiments; values are reported as the means ± SEMs; ***p < 0.0001 or **p < 0.001 for all samples versus scr-shRNA D4M cells, ^###^*p* < 0.0001 sh-TFEB + BCI-treated versus untreated sh-TFEB cells as determined by Student’s *t* test).

Using sh-Tfeb D4M and YUMM cells, we checked the activity of different enzymes of the glycolysis cascade and found impaired activity (Fig. 3A and Table 1) but not a change in the expression of phosphofructokinase (PFK), aldolase (ALDO), glyceraldehyde-3-phosphate dehydrogenase (GAPDH), enolase (ENO), PK or lactate dehydrogenase (LDH) (Supplementary Fig. S5A), resulting in a decrease in lactate production (Fig. 3B and Table 1). These alterations were probably related to the decrease in glucose uptake, as inferred by the reduction in glucose influx (Fig. 3C and Table 1), although the amount of GLUT-1 was not changed (Supplementary Fig. S5B) (the relative fold change in *Glut-1* in sh-Tfeb D4M cells compared with the expression in scr-shRNA D4M cells after normalisation to the level of the housekeeping gene *Tbp* was 1.0 ± 0.19; the value is reported as the means ± SEMs; p = ns, as determined by Student’s *t* test; *n* = 3 independent experiments).

**Table 1:** Analysis of metabolic pathways in melanoma YUMM cells after Tfeb silencing.

*Glycolysis*								
	Glucose uptake	PFK	Aldolase	GAPDH	Enolase	Pyruvate kinase	LDH	Lactate
	pmol/min/mg prot	nmol NAD/min/mg prot	nmol NAD/min/mg prot	nmol NADH/min/mg prot	nmol NAD/min/mg prot	nmol NAD/min/mg prot	nmol NAD/min/mg prot	nmol/mg prot
scr-shRNA YUMM 4.1	10.33 ± 0.5	2.59 ± 0.1	3.49 ± 0.1	11.87 ± 0.8	16.20 ± 0.8	2.19 ± 0.1	12.69 ± 0.3	251.49 ± 10.6
sh-Tfeb YUMM 4.1	7.53 ± 0.3**	0.86 ± 0.05***	1.42 ± 0.1***	5.77 ± 0.6**	7.33 ± 0.4***	1.18 ± 0.1***	7.6 ± 0.5***	121.59 ± 4.2***
scr-shRNA YUMM 3.3	11.72 ± 0.4	2.50 ± 0.1	3.08 ± 0.1	9,07 ± 0.6	12.28 ± 0.5	1.80 ± 0.1	10.41 ± 0.4	207.23 ± 3.3
sh-Tfeb YUMM 3.3	7.64 ± 0.61**	1.07 ± 0.05***	1.47 ± 0.08***	5.21 ± 0.25**	6.99 ± 0.3***	0.67 ± 0.03***	5.33 ± 0.4***	125.14 ± 8.07***
scr-shRNA YUMM 1.7	8.08 ± 0.3	1.84 ± 0.1	2.79 ± 0.1	8.68 ± 0.7	9.24 ± 0.5	1.52 ± 0.1	9.26 ± 0.2	182.95 ± 7.7
sh-Tfeb YUMM 1.7	4.6 ± 0.4**	0.76 ± 0.1***	0.96 ± 0.02***	3.02 ± 0.3***	4.58 ± 0.6**	0.53 ± 0.04**	3.75 ± 0.3***	93.14 ± 3***

*TCA cycle*							
	Pyruvate DH	Citrate synthase	Aconitase	Isocitrate DH	αketoglutarate DH	Succinate DH	TCA cycle flux
	nmol NADH/min/mg prot	nmol citrate/min/mg prot	nmol isocitrate/min/mg prot	nmol NADH/min/mg prot	nmol NADH/min/mg prot	nmol FADH2/min/mg prot	nmol CO2/h/mh prot
scr-shRNA YUMM 4.1	2.22 ± 0.1	0.9 ± 0.1	0.27 ± 0.02	1.04 ± 0.04	0.58 ± 0.02	0.85 ± 0.04	3.77 ± 0.1
sh-Tfeb YUMM 4.1	1.91 ± 0.04*	0.39 ± 0.1**	0.17 ± 0.03*	0.87 ± 0.1*	0.29 ± 0.1**	0.37 ± 0.2***	2.03 ± 0.6***
scr-shRNA YUMM 3.3	2.15 ± 0.1	1.05 ± 0.1	0.29 ± 0.03	1.5 ± 0.1	0.73 ± 0.04	0.82 ± 0.1	3.08 ± 0.1
sh-Tfeb YUMM 3.3	1.84 ± 0.1*	0.58 ± 0.1**	0.17 ± 0.02*	1.23 ± 0.1*	0.48 ± 0.03**	0.56 ± 0.05*	1.94 ± 0.1**
scr-shRNA YUMM 1.7	2.53 ± 0.2	1.40 ± 0.04	0.49 ± 0.04	1.61 ± 0.08	0.92 ± 0.05	1.14 ± 0.1	4.95 ± 0.4
sh-Tfeb YUMM 1.7	2.29 ± 0.2*	0.54 ± 0.05***	0.31 ± 0.2**	1.12 ± 0.1**	0.61 ± 0.02**	0.56 ± 0.05**	1.31 ± 0.12***

*Glutaminolysis*				
	GLS	GLU-DH	GOT	GS
	µmol NADH/min/mg prot	µmol NADH/min/mg prot	nmol Glu/min/mg prot	nmol/mg prot
scr-shRNA YUMM 4.1	8.64 ± 0.4	2.16 ± 0.03	33.95 ± 1.26	2.85 ± 0.1
sh-Tfeb YUMM 4.1	4.35 ± 0.2***	2.3 ± 0.04	34.68 ± 2.3	1.36 ± 0.05***
scr-shRNA YUMM 3.3	6.07 ± 0.2	2.6 ± 0.2	33.23 ± 1.01	2.84 ± 0.15
sh-Tfeb YUMM 3.3	3,91 ± 0.2**	2.58 ± 0.1	36.17 ± 1.8	1.17 ± 0.1***
scr-shRNA YUMM 1.7	6.14 ± 0.4	1.47 ± 0.1	32.63 ± 0.7	2.35 ± 0.3
sh-Tfeb YUMM 1.7	2.03 ± 0.1***	1.28 ± 0.1	33.53 ± 2.42	1.06 ± 0.04**

OXPHOS						Mitochondrial depolarisation/oxidative damages		
	Complex I	Complex II	Complex III	Complex IV	Mitochondrial ATP synthesis	mPTP	TBARS	SOD2
	nmol NAD/min/mg prot	nmol cit c/min/mg prot	nmol red cit c/min/mg prot	nmol ox cit c/min/mg prot	nmol/mg prot	dRFU/min/mg prot	nmol/mg prot	µmol red cit c/min/mg prot
scr-shRNA YUMM 4.1	0.48 ± 0.04	0.74 ± 0.03	0.33 ± 0.03	0.59 ± 0.05	41.81 ± 2	0.84 ± 0.06	67.54 ± 5.7	0.93 ± 0.05
sh-Tfeb YUMM 4.1	0.28 ± 0.03**	0.36 ± 0.03**	0.14 ± 0.02**	0.3 ± 0.01**	24.02 ± 2.1**	1.24 ± 0.07**	104.65 ± 3.7**	2.03 ± 0.06***
scr-shRNA YUMM 3.3	0.57 ± 0.04	0.84 ± 0.06	0.33 ± 0.03	0.72 ± 0.03	53.32 ± 2.3	0.75 ± 0.03	70.68 ± 1.5	1.71 ± 0.08
sh-Tfeb YUMM 3.3	0.4 ± 0.04*	0.41 ± 0.03**	0.22 ± 0.02*	0.45 ± 0.03**	24.65 ± 1.9***	1.64 ± 0.09***	114.89 ± 1.7***	2.7 ± 0.1**
scr-shRNA YUMM 1.7	0.70 ± 0.03	0.91 ± 0.02	0.47 ± 0.02	0.82 ± 0.04	75.06 ± 3.6	1.03 ± 0.05	96.47 ± 3.6	1.95 ± 0.04
sh-Tfeb YUMM 1.7	0.4 ± 0.04**	0.45 ± 0.03***	0.23 ± 0.03**	0.38 ± 0.04**	39.64 ± 2.66**	2.33 ± 0.1***	177.92 ± 5.68***	3.87 ± 0.06***

Lipid synthesis				
	Cholesterol synthesis	FPP	GGPP	Ubiquinone
	pmol/mg prot	pmol/mg prot	pmol/mg prot	pmol/mg prot
scr-shRNA YUMM 4.1	0.85 ± 0.04	0.15 ± 0.01	0.19 ± 0.01	0.48 ± 0.02
sh-Tfeb YUMM 4.1	0.36 ± 0.04***	0.05 ± 0.01***	0.12 ± 0.006**	0.23 ± 0.02**
scr-shRNA YUMM 3.3	0.81 ± 0.07	0.13 ± 0.01	0.24 ± 0.02	0.51 ± 0.01
sh-Tfeb YUMM 3.3	0.52 ± 0.04*	0.05 ± 0.001*	0.08 ± 0.02**	0.27 ± 0.02***
scr-shRNA YUMM 1.7	0.72 ± 0.05	0.16 ± 0.01	0.23 ± 0.02	0.73 ± 0.02
sh-Tfeb YUMM 1.7	0.35 ± 0.01**	0.08 ± 0.02**	0.08 ± 0.02***	0.3 ± 0.02**

(*n* = 3 independent experiments; values as means ± SEMs; ***p < 0.0001,**p < 0.001 and *p < 0.01 for sh-Tfeb versus scr-shRNA-YUMM, as determined by Student’s *t* test).

We focused on PKM2, the most highly expressed PK isoform in cancer. PKM2 directs glycolytic flux towards pyruvate or the accumulation of intermediates that are consumed during serine biosynthesis and the pentose phosphate pathway [24–27].

After *Tfeb* silencing in D4M cells, we found that total PKM2 expression was unchanged (Fig. 3D, Supplementary Fig. S4D and Supplementary Fig. S5A), while a reduction in the cellular and nuclear levels of pSer37-PKM2 was evident (Fig. 3D and Supplementary Fig. S4D).

As observed for p-ERK1/2 levels (Fig. 1D), the treatment of sh-Tfeb D4M cells with the DUSP-1 inhibitor BCI rescued the amount of pSer37-PKM2 (Fig. 3D and Supplementary Fig. S4D) and its nuclear localisation (Fig. 3D), suggesting putative ERK1/2-DUSP-1 pathway involvement in PKM2 activity as a transcriptional regulator.

### TFEB influences TCA flux

**Fig. 4:**
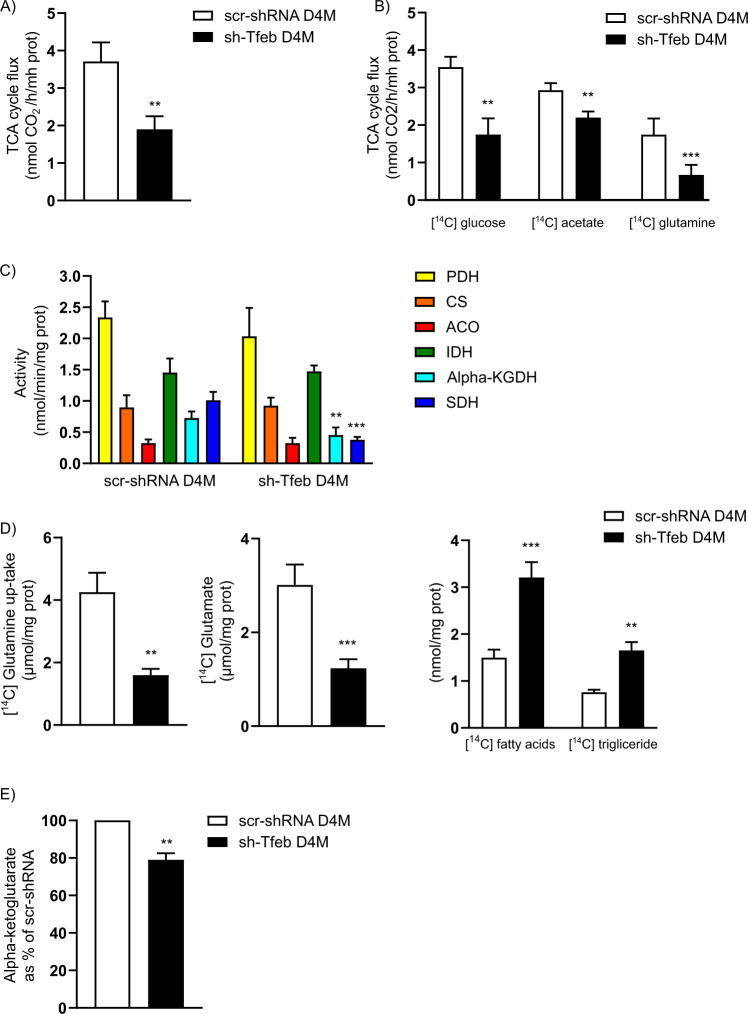
*Tfeb* silencing induces reverse TCA flux. **A** The bar graph shows the rate of TCA cycle flux in scr-shRNA and sh-Tfeb D4M cells (*n* = 3 independent experiments; values are reported as the means ± SEMs; **p < 0.001 for sh-Tfeb versus scr-shRNA D4M cells, as determined by Student’s *t* test). **B** The bar graph shows the rate of TCA cycle flux in scr-shRNA and sh-Tfeb D4M cells after cell supplementation with [^14^C] glucose, [^14^C] acetate or [^14^C] glutamine (*n* = 3 independent experiments; values are reported as the means ± SEMs; ***p < 0.0001 or **p < 0.001 for sh-Tfeb versus scr-shRNA D4M cells, as determined by Student’s *t* test). **C** The bar graph shows the activity of enzymes involved in TCA cycle flux in scr-shRNA and sh-Tfeb D4M cells (the activity of PDH, IDH and alpha-KGDH was measured as nmol NADH/min/mg protein; the activity of CS and ACO was measured as nmol citrate or isocitrate/min/mg protein; the activity of SDH was measured as nmol FADH2/min/mg protein) (*n* = 3 independent experiments; values are reported as the means ± SEMs; ***p < 0.0001 and **p < 0.001 for sh-Tfeb versus scr-shRNA D4M cells, as determined by Student’s *t* test). **D** The bar graphs show the amount of [^14^C] glutamine up-take, and [^14^C] glutamate, [^14^C] FA and [^14^C] triglyceride levels after scr-shRNA and sh-Tfeb D4M cells treatment with [^14^C] glutamine (*n* = 3 independent experiments; values are reported as the means ± SEMs; ***p < 0.0001 and **p < 0.001 for sh-Tfeb versus scr-shRNA D4M cells, as determined by Student’s *t* test). **E** The bar graph shows the level of alpha-ketoglutarate in scr-shRNA and sh-Tfeb D4M cells (*n* = 3 independent experiments; values are reported as the means ± SEMs; **p < 0.001 for sh-Tfeb versus scr-shRNA D4M cells, as determined by Student’s *t* test).

Similar to our findings showing glycolytic reduction, we evaluated an impairment in TCA flux (Fig. 4A and Table 1) in all *Tfeb* silenced melanoma cells. Although sh-Tfeb D4M cells were characterised by an increase in cellular acetate concentration (Supplementary Fig. S5C), the TCA flux sustained by exogenous implementation of [^14^C]-acetate or [^14^C]-glucose was reduced (Fig. 4B). Glutamine-mediated TCA flux was also decreased (Fig. 4B), suggesting that *Tfeb* silencing correlated with an inhibition of both glucose- and glutamine-fuelled TCA flux.

Interestingly, in sh-Tfeb D4M and YUMM cells, we detected unchanged activity for the enzymes involved in the first part of the TCA cycle, such as pyruvate dehydrogenase (PDH), citrate synthase (CS), aconitase (ACO), and isocitrate dehydrogenase (IDH), while the activity of the proteins involved the second part of the TCA cycle sustained by glutamate such as alpha-ketoglutarate (alpha-KGDH) and succinate dehydrogenase (SDH) was clearly blocked (Fig. 4C and Table 1). Notably, the expression of different enzymes involved in this pathway was unchanged (Supplementary Fig. S5A).

We investigated glutamine flux and metabolism after [^14^C]-glutamine cell loading. In sh-Tfeb D4M cells, we observed a reduction in [^14^C]-glutamine uptake (Fig. 4D) and [^14^C] glutamate concentration (Fig. 4D), which was not sustained by a defect of the expression of glutamine transporter SLC1A5 (Supplementary Fig. S5B). (the relative fold change of *Slc1a5* in sh-Tfeb D4M cells compared with that in scr-shRNA D4M cells after level normalisation to that of the housekeeping gene *Tbp* was 1.1 ± 0.17; the value is reported as the means ± SEMs; p = ns, as determined by Student’s *t* test; *n* = 3 independent experiments).

*Tfeb* silencing in all melanoma cells reduced the catalytic activity of glutaminase (GLS) without modifying its expression (Supplementary Fig. S5D, Supplementary Fig. S5E and Table 1), but it had no effect on glutamic dehydrogenase (GLU DH) activity (Supplementary Fig. S5D and Table 1). In sh-Tfeb D4M cells the level of alpha-ketoglutarate was reduced (Fig. 4E), likely reflecting the lower glutaminolytic flux.

We also considered the regulation of glutamine synthesis and demonstrated that *Tfeb* silencing reduced glutamine synthase (GS) activity (Supplementary Fig. S5F and Table 1) without affecting its expression (Supplementary Fig. S5G).

In sh-Tfeb D4M and YUMM cells, the expression and activity of IDH (Supplementary Fig. S5A, Fig. 4C and Table 1) was not modified, while after [^14^C]-glutamine supplementation the synthesis of [^14^C]-fatty acid (FA) (Fig. 4D) and [^14^C]-triglyceride (Fig. 4D) was increased, suggesting that glutamine was metabolised via reductive carboxylation rather than glutaminolysis.

### TFEB regulates oxidative phosphorylation (OXPHOS)

**Fig. 5:**
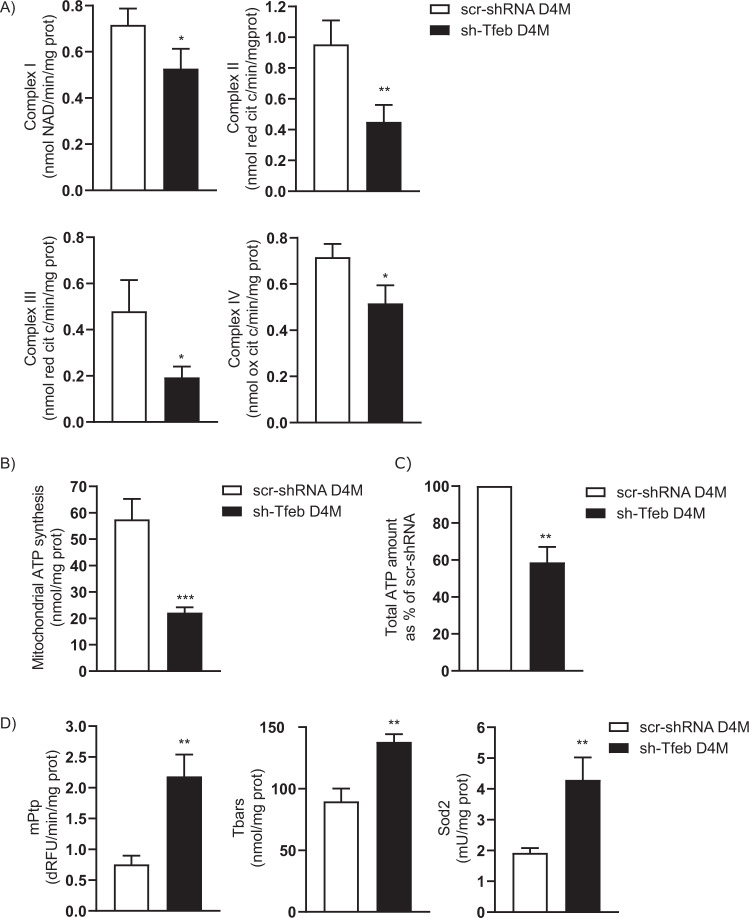
*Tfeb* silencing leads to inhibited mitochondrial ATP synthesis and increased mitochondrial oxidative stress. **A** The bar graphs show the level of mitochondrial complex activity in scr-shRNA and sh-Tfeb D4M cells (*n* = 3 independent experiments; values are reported as the means ± SEMs; **p < 0.001 and *p < 0.01 for sh-Tfeb versus scr-shRNA D4M cells, as determined by Student’s *t* test). **B**, **C** The bar graphs show the rate of mitochondrial ATP synthesis and the cellular ATP levels in scr-shRNA and sh-Tfeb D4M cells (*n* = 3 independent experiments; values are reported as the means ± SEMs; ***p < 0.0001 and **p < 0.001 for sh-Tfeb versus scr-shRNA D4M cells, as determined by Student’s *t* test). **D** The bar graphs show the quantification of the mPtp, Tbars and Sod2 levels in scr-shRNA and sh-Tfeb D4M cells (*n* = 3 independent experiments; values are reported as the means ± SEMs; **p < 0.001 for sh-Tfeb versus scr-shRNA D4M cells, as determined by Student’s *t* test).

Sh-Tfeb D4M and YUMM cells were characterised by a reduction in OXPHOS and ATP synthesis compared to scr-shRNA cells (Fig. 5A–C and Table 1).

Mitochondrial function seemed to be dampened after *Tfeb* silencing, as suggested by the increase in the oxidative and damage indexes, i.e. the frequency of mitochondrial transition pore (mPtp) opening, the level of thiobarbituric acid reactive substances (Tbars) and the activity of superoxide dismutase 2 (Sod2) (Fig. 5D and Table 1).

### TFEB controls cholesterol synthesis

**Fig. 6:**
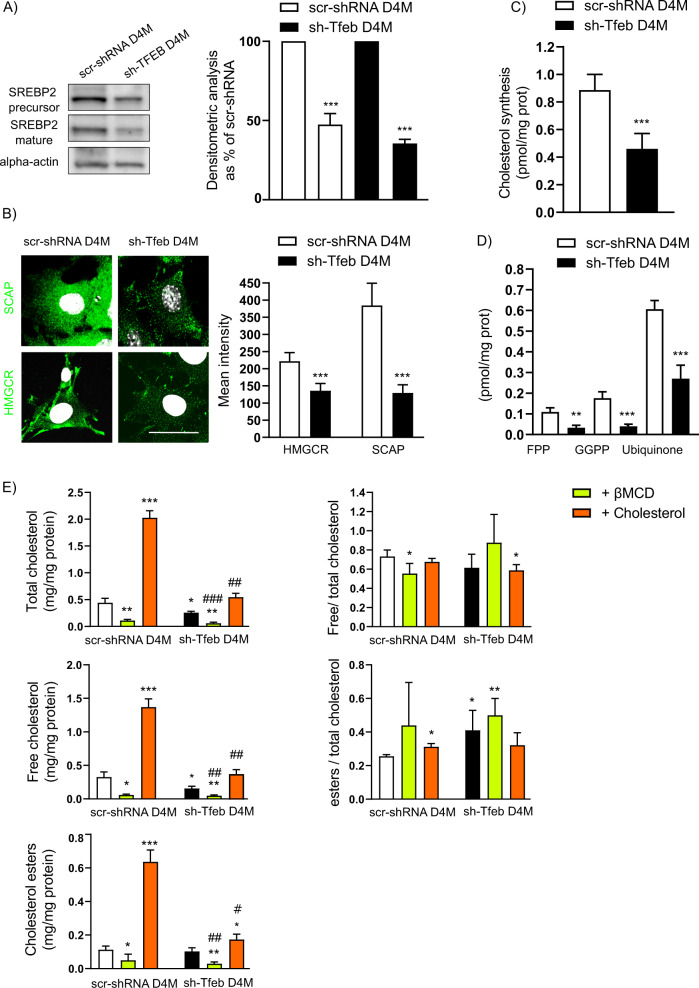
*Tfeb* silencing leads to inhibited cholesterol synthesis. **A**, **B** Representative western blots and confocal microscopy analysis (scale bar: 25 µm) show the expression level of the SREBP2 precursor and mature proteins and the SCAP protein level in scr-shRNA and sh-Tfeb D4M cells. The bar graphs show the densitometry results and the mean intensity of the SREBP2 precursor and mature protein, SCAP and HMGCR signals (*n* = 3 independent experiments for western blotting; *n* = 15 cells per condition from three different experiments for immunofluorescence measures; the means ± SEMs; ***p < 0.0001 for sh-Tfeb versus scr-shRNA D4M cells, as determined by Student’s *t* test). **C**, **D** The bar graphs show the levels of cholesterol synthesis, FPP, GGPP, and ubiquinone in scr-shRNA and sh-Tfeb D4M cells (*n* = 3 independent experiments; values are reported as the means ± SEMs; ***p < 0.0001 and **p < 0.001 for sh-Tfeb versus scr-shRNA D4M cells, as determined by Student’s *t* test). **E** The bar graphs show the levels of total, free and ester cholesterol and the ratio between free cholesterol level/total cholesterol levels or ester/total cholesterol levels in scr-shRNA and sh-Tfeb D4M cells after treatment with βMCD (5 mM, 1 h) and supplementation with cholesterol (10 µg/ml, 24 h) (*n* = 3 independent experiments; values are reported as the means ± SEMs; ***p < 0.0001, **p < 0.001 and *p < 0.01 for all samples versus scr-shRNA D4M cells; ^###^*p* < 0.0001, ^##^*p* < 0.001 and ^#^*p* < 0.01 for treated sh-Tfeb cells versus untreated sh-Tfeb D4M cells as determined by Student’s *t* test).

As reported in endothelial cells [35] in sh-Tfeb D4M and YUMM cells, the synthesis of cholesterol; the upstream isoprenoid metabolites farnesyl pyrophosphate (FPP) and geranylgeranyl pyrophosphate (GGPP); and the isoprenoid-derivative ubiquinone (Fig. 6C–E and Table 1) was dampened. This effect was connected with the inhibition of transcription and protein expression of the major regulators of cholesterol homeostasis, namely, SCAP, SREBP2 and HMGCR (Supplementary Fig S5H, Fig. 6A, B).

Moreover, the free/total cholesterol ratio (Fig. 6E) suggested that free cholesterol levels were reduced in sh-Tfeb D4M cells as a consequence of the inhibited cholesterol synthesis. In contrast, the increase in the ratio of ester/total cholesterol levels suggested cholesteryl ester accumulation (Fig. 6E).

The structure and the function of GLUT-1 and SLC1A5 are affected by the membrane cholesterol level [65–68]. We thus evaluated in sh-Tfeb D4M cells if the addiction of exogenous cholesterol to the level measured in scr-shRNA D4M cells was able to recover the altered metabolic activities induced by *Tfeb* silencing.

Upon depletion of endogenous cholesterol by beta methyl cyclodextrin (βMCD) (5 mM, 1 h), D4M cells were treated with exogenous cholesterol (10 µg/ml, 24 h), capable of restoring total, free and esters cholesterol levels in sh-Tfeb cells to those observed in scr-shRNA cells (Fig. 6E). Treatment of the scr-shRNA and sh-TFEB cells with βMCD induced a significant reduction in glucose and glutamine flux, as well as glycolysis, lactate production and glutaminolysis rates; moreover, it reversed TCA cycle activity and reduced the OXPHOS, and ATP synthesis rates (Table 2). The metabolite flux rates and activity of the related metabolic pathways were rescued by the addition of exogenous cholesterol (Table 2) in both scr-shRNA and sh-Tfeb D4M cells.

**Table 2:** Analysis of metabolic pathways in melanoma D4M cells after Tfeb silencing and cholesterol supplementation.

*Glycolysis*								
	Glucose uptake	PFK	Aldolase	GAPDH	Enolase	Pyruvate kinase	LDH	Lactate
	(pmol/mg prot)	nmol NAD/min/mg prot	nmol NAD/min/mg prot	nmol NADH/min/mg prot	nmol NAD/min/mg prot	nmol NAD/min/mg prot	nmol NAD/min/mg prot	nmol/mg prot
scr-shRNA D4M	11.29 ± 0.6	3.18 ± 0.07	3.14 ± 0.05	14.96 ± 0.36	15.06 ± 1.23	1.73 ± 0.12	10.79 ± 0.28	231.06 ± 7.72
sh-Tfeb D4M	6.66 ± 0.4	1.15 ± 0.05	1.13 ± 0.17	5.27 ± 0.35	5.63 ± 0.3	0.59 ± 0.05	5.95 ± 0.3	111.95 ± 2.8
scr-shRNA D4M + βmcd	8.34 ± 0.7§§	1.89 ± 0.15§§§	2.56 ± 0.24§	10.64 ± 0.32§§§	8.80 ± 0.18§§§	1.18 ± 0.13§	7.53 ± 0.3§§§	150.89 ± 17.01§
sh-Tfeb D4M + βmcd	4.58 ± 0.27°	0.94 ± 0.04°	0.69 ± 0.04°	3.66 ± 0.31°	3.05 ± 0.37°	0.36 ± 0.04°	3.89 ± 0.13°°°	97.46 ± 6.69
scr-shRNA D4M + cholesterol	14.93 ± 0.3	3.65 ± 0.1	4.12 ± 0.07	17.62 ± 0.6	16.37 ± 0.9	1.87 ± 0.2	12.99 ± 0.6	288.22 ± 2.6
sh-Tfeb D4M + cholesterol	8.12 ± 0.1 #,**	1.44 ± 0.04 ##,***	1.66 ± 0.1 #,***	6.90 ± 0.2 ##,***	6.35 ± 0.1 #,**	0.84 ± 0.06 #,**	7.27 ± 0.5 #,**	140.98 ± 9.01 #,***

*TCA cycle*							+[^14^C]glucose	+[^14^C]glutamine
	Pyruvate DH	Citrate synthase	Aconitase	Isocitrate DH	αketoglutarate DH	Succinate DH	TCA cycle flux	TCA cycle flux
	nmol NADH/min/mg prot	nmol citrate/min/mg prot	nmol isocitrate/min/mg prot	nmol NADH/min/mg prot	nmol NADH/min/mg prot	nmol FADH2/min/mg prot	nmol CO2/h/mh prot	nmol CO2/h/mh prot
scr-shRNA D4M	2.3 ± 0.1	0.85 ± 0.05	0.32 ± 0.03	1.35 ± 0.1	0.85 ± 0.05	0.86 ± 0.03	3.13 ± 0.05	2.29 ± 0.1
sh-Tfeb D4M	1.93 ± 0.1	0.83 ± 0.01	0.17 ± 0.02	1.23 ± 0.1	0.33 ± 0.1	0.4 ± 0.04	1.84 ± 0.1	0.55 ± 0.1
scr-shRNA D4M + βmcd	2.03 ± 0.06§	0.68 ± 0.05§	0.27 ± 0.04	1.12 ± 0.06§§§	0.42 ± 0.06§§§	0.44 ± 0.06§§§	2.32 ± 0.09§§§	1.68 ± 0.13§
sh-Tfeb D4M + βmcd	1.97 ± 0.06	0.74 ± 0.05	0.24 ± 0.01°	1.02 ± 0.02	0.12 ± 0.02°	0.25 ± 0.03°	1.34 ± 0.05°	0.31 ± 0.03°
scr-shRNA D4M + cholesterol	2.32 ± 0.05	0.89 ± 0.04	0.35 ± 0.03	1.29 ± 0.1	1.65 ± 0.1	1.19 ± 0.1	3.45 ± 0.2	2.90 ± 0.1
sh-Tfeb D4M + cholesterol	2.06 ± 0.05#,*	0.88 ± 0.05	0.32 ± 0.02	1.18 ± 0.01	0.83 ± 0.04##	0.82 ± 0.04##	2.54 ± 0.02#,*	1.80 ± 0.1###,*

*Glutaminolysis*			Input	[^14^C] glutamine			
	GLS	GLU-DH	Intracellular recovery	[^14^C] glutamine	[^14^C] glutamate	[^14^C] FA	[^14^C] Trigliceride
	µmol NADH/min/mg prot	µmol NADH/min/mg prot		µmol/mg prot	µmol/mg prot	nmol/mg prot	nmol/mg prot
scr-shRNA D4M	8.06 ± 0.11	2.22 ± 0.1		4.64 ± 0.2	3.01 ± 0.2	1.5 ± 0.1	0.76 ± 0.03
sh-Tfeb D4M	3.21 ± 0.3	2.26 ± 0.1		1.49 ± 0.1	1.23 ± 0.1	3.21 ± 0.2	1.65 ± 0.1
scr-shRNA D4M + βmcd	4.58 ± 0.28§§§	1.83 ± 0.04§§§		2.61 ± 0.28§§§	1.58 ± 0.06§§§	1.14 ± 0.06§§	0.74 ± 0.04
sh-Tfeb D4M + βmcd	1.37 ± 0.05°°°	1.76 ± 0.07°		0.73 ± 0.06°°°	0.57 ± 0.08°°°	2.33 ± 0.11°	1.64 ± 0.1
scr-shRNA D4M + cholesterol	13.43 ± 0.6	2.55 ± 0.1		6.61 ± 0.3	4.19 ± 0.03	0.77 ± 0.04	0.57 ± 0.03
sh-Tfeb D4M + cholesterol	6.38 ± 0.3###, **	2.47 ± 0.05*		4.23 ± 0.2###	2.95 ± 0.1###	1.32 ± 0.11###	0.89 ± 0.1##

OXPHOS					
	Complex I	Complex II	Complex III	Complex IV	Mitochondrial ATP synthesis
	nmol NAD/min/mg prot	nmol cit c/min/mg prot	nmol red cit c/min/mg prot	nmol ox cit c/min/mg prot	nmol/mg prot
scr-shRNA D4M	0.66 ± 0.03	0.95 ± 0.06	0.56 ± 0.05	0.71 ± 0.03	54.3 ± 2.02
sh-Tfeb D4M	0.4 ± 0.05	0.4 ± 0.04	0.22 ± 0.04	0.46 ± 0.04	23.3 ± 3.21
scr-shRNA D4M + βmcd	0.44 ± 0.05§	0.72 ± 0.02§	0.37 ± 0.03§	0.60 ± 0.04§	38.2 ± 2.04§§§
sh-Tfeb D4M + βmcd	0.19 ± 0.03°	0.27 ± 0.02°	0.13 ± 0.01°	0.34 ± 0.01°	13.7 ± 1.07°
scr-shRNA D4M + cholesterol	0.80 ± 0.06	1.19 ± 0.11	0.70 ± 0.03	0.86 ± 0.03	56.7 ± 1.47
sh-Tfeb D4M + cholesterol	0.65 ± 0.03#	0.79 ± 0.06##	0.49 ± 0.05##	0.65 ± 0.03#	44.2 ± 4.76#

(*n* = 3 independent experiments; values as means ± SEMs; §§§*p* < 0.0001,§§*p* < 0.001 and §§*p* < 0.01 for sh-RNA + βmcd versus scr-shRNA D4M, °°°*p* < 0.0001 and °*p* < 0.01 sh-Tfeb + βmcd versus sh-Tfeb D4M; ***p < 0.0001,**p < 0.001 and *p < 0.01 for sh-Tfeb + cholesterol versus scr-shRNA D4M, ^###^*p* < 0.0001, ^##^*p* < 0.001 and ^#^*p* < 0.01 sh-Tfeb + cholesterol versus sh-Tfeb D4M as determined by Student’s *t* test).

In particular, in sh-Tfeb D4M cells supplemented with cholesterol and [^14^C]-glutamine, we detected complete reactivation of [^14^C]-glutamine uptake and normalisation of its metabolism via glutaminolysis, not reductive carboxylation, as evidenced by the normalisation of [^14^C]-glutamate, [^14^C]-FA and [^14^C]-triglyceride levels, which were similar to those in the scr-shRNA D4M cells (Table 2).

In *Tfeb* silenced D4M cells, after cholesterol addition, mitochondrial OXPHOS and ATP synthesis were also rescued (Table 2).

### The combined inhibitory effects of *Tfeb* silencing and pharmacological treatment of the BRAFV600E mutant

We verified the effects of the combination of *Tfeb* silencing and PLX4720, a 7-azaindole derivative that inhibits BRAFV600E activity [69].

PLX4720 treatment reduced the growth of scr-shRNA D4M cells (Supplementary Fig. S6A), the phosphorylation of ERK1/2 and the expression of Cyclin D1 and PCNA (Supplementary Fig. S6B, C) but did not modify TFEB expression (Supplementary Fig. S6B, C). Furthermore, PLX4720 treatment of scr-shRNA D4M cells reduced the percentage of cells in the S phase of the cell cycle (Supplementary Fig. S6D) and the ATP synthesis rate (Supplementary Fig. S6E).

Interestingly, *Tfeb* silencing exerted effects similar to those of PLX4720 treatment (Supplementary Fig. S6A–C), and the combination of *Tfeb* silencing and PLX4720 treatment exerted an addictive effect compared to that of PLX4720 treatment alone (Supplementary Fig. S6A–E).

### TFEB silencing dampens melanoma growth

**Fig. 7:**
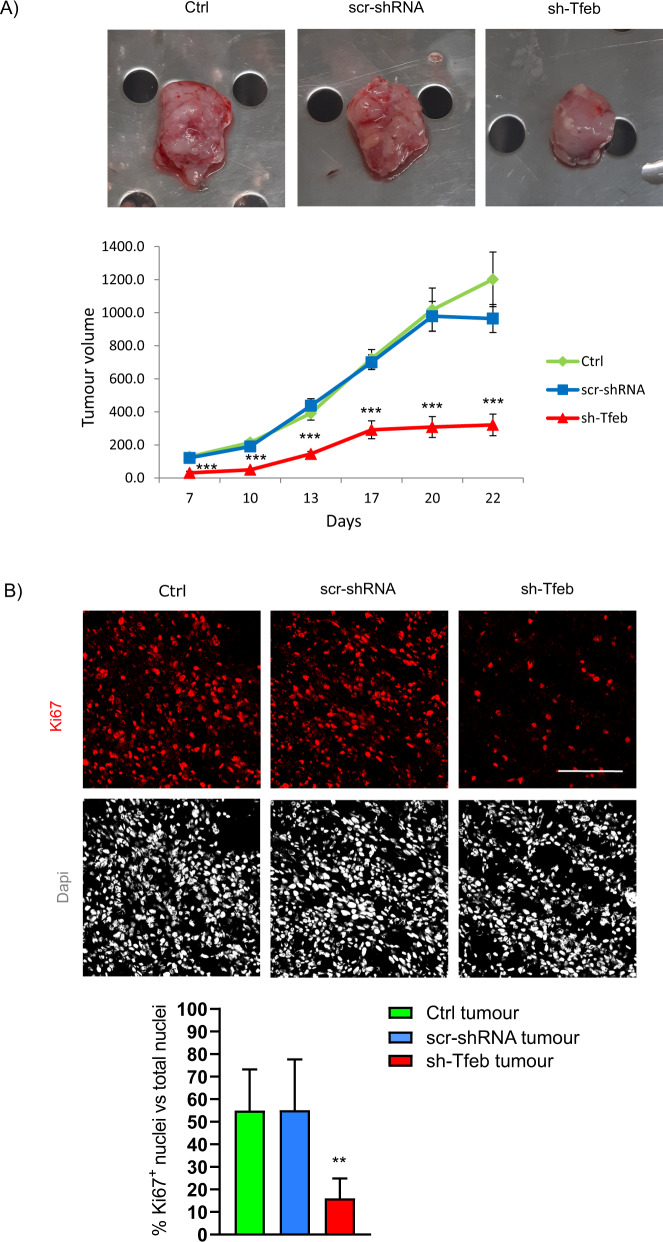
*Tfeb* silencing leads to inhibited melanoma growth. **A** Representative image show Ctrl, scr-shRNA and sh-Tfeb D4M tumours explanted from mice 22 days after subcutaneous injection of 1 × 10^6^ the respective cells and the relative growth curve (*n* = 5 ctrl tumours, *n* = 9 scr-shRNA and sh-Tfeb D4M tumours; values are reported as the means ± SEMs; ***p < 0.0001 for sh-Tfeb versus scr-shRNA D4M tumours, as determined by Student’s *t* test). **B** Confocal microscopy analysis of Ki-67 expression in Ctrl, scr-shRNA and sh-Tfeb D4M tumours after incubation with an anti-Ki-67 antibody and Dapi (scale bar: 25 µm). The bar graph shows the quantification of Ki-67^+^ nuclei as a percentage of total nuclei (*n* = 15 images obtained from three different tumours for each condition; values are reported as the means ± SEMs; **p < 0.0001 sh-Tfeb versus scr-shRNA D4M tumours, as determined by Student’s *t* test).

On the basis of the in vitro data, we evaluated whether *Tfeb* silencing leads to reduced melanoma growth in mice. C57BL/6 mice were subcutaneously injected with ctrl D4M cells or scr-shRNA or sh-Tfeb D4M cells and euthanized 22 days later (Supplementary Fig. S7). Figure 7A clearly shows that sh-Tfeb D4M tumours grew slower and to a lower size, with a volume reduction of 66.7% at the endpoint. The analysis of Ki-67^+^ nuclei in D4M tumours indicated that the percentage of proliferating cells decreased in the sh-Tfeb D4M tumour samples compared to controls (Fig. 7B).

**Fig. 8:**
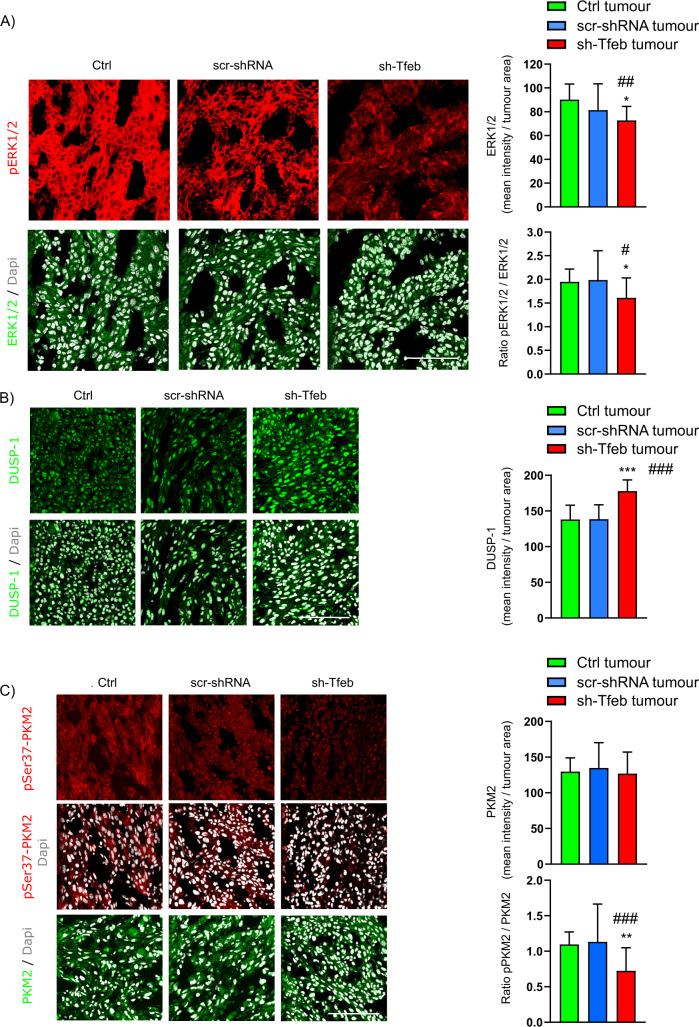
*Tfeb* silencing inhibits ERK1/2 signalling and PKM2 phosphorylation and up-regulate DUSP-1 in melanoma tumours. **A**–**C** Confocal microscopy analysis of the levels of phosphorylated- and total ERK1/2, DUSP-1 and phosphorylated- and total PKM2 expression in Ctrl, scr-shRNA and sh-Tfeb D4M tumours after incubation with specific antibodies (scale bar: 25 µm). The bar graphs show the mean protein signal intensity/tumour area, the pERK1/2/ERK1/2 ratio and the pPKM2/PKM2 ratio (*n* = 15 images pooled from three different tumours for each condition; values are reported as the means ± SEMs; ***p < 0.0001, **p < 0.001, *p < 0.01 for sh-Tfeb versus scr-shRNA D4M tumours, and ^###^*p* < 0.0001, ^##^*p* < 0.001 and ^#^*p* < 0.01 for sh-Tfeb versus Ctrl D4M tumours as determined by Student’s *t* test).

According to the in vitro data, sh-Tfeb D4M tumours showed reduced ERK1/2 activation, which was reported as the ratio of p-ERK1/2/ERK1/2 and an increase in DUSP-1 expression (Fig. 8A, B). Moreover, sh-Tfeb D4M tumours showed a decrease in p-Ser37-PKM2 level (Fig. 8C).

Compared to control D4M tumours, in sh-Tfeb D4M tumours, glycolysis (Supplementary Fig. S8A), TCA flux (Supplementary Fig. S8B), glutaminolysis and glutamine synthesis (Supplementary Fig. S8C, D), OXPHOS and mitochondrial ATP synthesis (Supplementary Fig. S8E and Table 3) were all inhibited, and mitochondrial damage and dysfunction malfunction were increased (Table 3).

**Table 3:** Analysis of OXPHOS and mitochondrial damage in melanoma tumour after Tfeb silencing.

OXPHOS				MITOCHONDRIAL DAMAGE		
	Complex I	Complex II	Complex III	Complex IV	TBARS	SOD2
	nmol NAD/min/mg prot	nmol cit c/min/mg prot	nmol red cit c/min/mg prot	nmol ox cit c/min/mg prot	nmol/mg prot	µmol red cit c/min/mg prot
ctrl tumour	0.75 ± 0.1	0.76 ± 0.1	0.68 ± 0.05	0.91 ± 0.1	51.93 ± 6.2	1.45 ± 0.2
scr-shRNA tumour	0.67 ± 0.04	0.75 ± 0.1	0.65 ± 0.04	0.78 ± 0.1	50.39 ± 5.9	1.88 ± 0.1
sh-Tfeb tumour	0.33 ± 0.05 *** ###	0.26 ± 0.03 *** ###	0.24 ± 0.03 *** ###	0.28 ± 0.03 *** ###	136.06 ± 13.8 *** ###	4.34 ± 0.3 *** ###

(*n* = 5 ctrl tumour, *n* = 9 scr-shRNA and sh-Tfeb tumour; values as means ± SEMs; ***p < 0.0001 sh-Tfeb versus scr-shRNA-tumour, ^###^*p* < 0.0001 sh-Tfeb versus ctrl-tumour as determined by Student’s *t* test).

In parallel, sh-Tfeb D4M tumours compared Ctrl D4M tumours were characterised by a reduced cholesterol level (Supplementary Fig. S8F).

## Discussion

In the present study, we demonstrate a key role for TFEB in melanoma that is independent of BRAF mutation, which is a genetic trait that characterises ~52% of human melanomas [2]. Our data suggest that this transcription factor contributes to the control of the cell cycle and the fulfilment of metabolic demands to support cell growth. The silencing of *Tfeb* in murine BRAF-WT and BRAF-mutated cell lines induced a proliferation blockade both in vitro and in vivo and led to reduced glycolysis, TCA cycle, OXPHOS and cholesterol synthesis activity.

We identified three putative and partially interconnected mechanisms sustaining the observed phenotype: (1) regulation of the cell cycle by its direct control of CDK4 expression; (2) modulation of the MAPK pathway via its direct repression of DUSP-1 expression, which leads to the dephosphorylation of p-ERK1/2; and (3) control of cholesterol synthesis by its indirect activity on the expression of SCAP, SREBP2 and HMGCR.

Melanoma, similar to other tumours, is characterised by lineage-exclusive mechanisms that coordinate metabolism and cell proliferation, two events that affect each other, showing continuous reciprocal modulation. The cross-talk among metabolism- and proliferation-related components adds complexity to the effects of specific mutations or alterations of a single pathway and is mediated by the concomitant actions of more than one factor [11, 70–72].

Therefore, a comprehensive analysis of the effect of TFEB on melanoma cells requires understanding of how the identified mechanisms fit into the general transcriptional landscape, which is mediated by TFEB itself, and how the participation of TFEB in other gene networks is influenced by somatic driver mutations [73].

TFEB silencing leads to decreased expression of *Cdk4*, as previously reported [39, 40], increased expression of the DUSP-1 protein, and a reduction in ERK1/2 phosphorylation and Cyclin D1 expression.

ERK1/2 activates a cascade of transcription factors that ultimately modulate genetic programmes, including the expression of *Ccnd1* [9, 10, 13, 14]. Moreover, ERK1/2 favours the interplay between Cyclin D1 and CDK4 [9, 10, 13, 14], resulting in the G1-S phase cell cycle transition.

In agreement with these data, in *Tfeb* silenced cells, DUSP-1 mediated p-ERK1/2 dephosphorylation, resulting in decreased *Ccnd1* transcription and expression, supporting the idea that it controlled ERK1/2, which ultimately modulates Cyclin D1 expression. Notably, our experiments showed the complete rescue of ERK1/2 phosphorylation after DUSP-1 activity inhibition but only a partial recovery of Cyclin D1 expression, suggesting that other unknown mechanisms might be involved in *Tfeb* silencing of melanoma cells.

ERK1/2 was described as an inhibitor of TFEB nuclear translocation via TFEB phosphorylation at S142 [29, 30]. Notably, this prior study showed that TFEB controlled ERK activity via the transcriptional modulation of DUSP-1.

DUSP genes are early genes that encode proteins coordinating a complex array of cellular functions [53–56], and these activities are ultimately regulated at the mRNA and protein levels [53–56] also in cancer [74]. Overexpression of DUSP-1 has been reported to decrease the growth rate, the invasion and migration capacities of non-small cell lung cancer cells and thus inhibit bone metastasis [75]. In melanoma patients, the low expression of DUSP-1 has been associated with worsened overall survival [76], probably mediated by the reduced dephosphorylation rate of several MAPKs and consequent increase in the proliferation rate [56]. In general, DUSP dysregulation has been associated with poor prognoses and resistance to therapeutic regimens in several cancers [74, 77–80].

Our data further indicate that TFEB activities regulating melanoma cell proliferation are not limited to effects on the cell cycle but extend to the control of metabolic pathways.

Recent evidence indicates that metabolism is not required merely to supply ATP and biomass to proliferating cells; in contrast, but metabolic enzymes and cell cycle regulators undergo reciprocal activation [81]. Alterations in metabolic pathways have been extensively described in melanoma and have been reported to rely partially on the dysregulated MAPK pathway [11, 82]. In particular, BRAF mutations and MAPK hyperactivation induce melanoma cell proliferation, mitochondrial alterations and drive metabolism switching from OXPHOS to glycolysis and support high metabolic flexibility [70, 71].

When expressed in normal cells, BRAFV600E enhances the expression of pyruvate dehydrogenase phosphatase, favouring pyruvate consumption in the TCA cycle and sustaining oncogene-induced senescence. This process is reversed in melanoma cells: pyruvate dehydrogenase kinase is typically upregulated, suppressing pyruvate dehydrogenase and decoupling glycolysis from mitochondrial respiration, thus contributing to tumorigenesis [11].

The TCA cycle is also involved in tumour formation and progression not only through its metabolic activity but through the TCA metabolites that support epigenetic modifications or posttranslational protein modifications, contributing to the initiation and progression of carcinogenesis [83].

Interestingly, melanoma TCA is not replenished by precursors through the anaplerotic enzyme pyruvate carboxylase but glutamine is the major source [84]. After entering a cell through the SLC1A5 receptor, glutamine is metabolised via classical glutaminolysis but also through reductive carboxylation, increasing the amount of citrate available for consumption in FA synthesis. Mutations in some of the genes involved in these processes occur in melanoma and favour its progression [84].

When TFEB is downregulated, melanoma cells respond by decreasing the uptake of glucose and glutamine; glycolytic and TCA anaplerotic flux; OXPHOS; and the synthesis of lactate, ATP and cholesterol. Interestingly, *Tfeb* silenced cells are characterised by reverse reductive TCA.

The reduction in these metabolic pathway rates was not mediated by alteration to enzyme expression but by a reduction in their activity, indicating that TFEB largely acts on metabolism in a manner independent of its direct control of gene transcription. However, our data provide precise insights into cholesterol synthesis, showing that the expression of *Scap*, *Srebf2* and *Hmcgr* was diminished in silenced melanoma cells.

To reconcile these observations consistent with an operational mechanism coordinated by TFEB, we primarily focused on the reduction in cholesterol synthesis and thus the reduced availability of cholesterol in cell membranes. Cholesterol is a key regulator of plasma membrane plasticity and rigidity, regulating the activities of membrane-embedded proteins [85].

The reduced uptake of glucose and glutamine in *Tfeb* silenced cells might be explained by the altered transporter function of GLUT-1 and SLC1A5 due to the decrease in plasma membrane cholesterol levels, as suggested by the unchanged levels of these proteins in these cells. In *Tfeb* silenced cells the rescue of cholesterol level similar to control cells led to the reactivation of glucose and glutamine uptake, the TCA cycle and mitochondrial functions, suggesting that GLUT-1 and SLC1A5 activity was regulated by TFEB via its control of cholesterol synthesis.

GLUT-1 is characterised by a cholesterol recognition/interaction amino acid consensus (CRAC) motif in its juxtamembrane fragment, and its transporter function is linked to cholesterol levels in the plasma membrane [67, 68]. Indeed, in many cell types, treatment with statins or methyl-β-cyclodextrin decreased glucose uptake by altering GLUT-1 activity [67, 68]. Similarly, cryo-EM structures of SLC1A5 revealed the presence of a CRAC motif [65, 66]. Cholesterol levels influence the initial transport rate of SLC1A5 but not its affinity (K_m_) for glutamine, and cholesterol probably induces conformational changes in the transporter. Moreover, glutamine transport has been shown to be reduced after cholesterol deprivation [65].

The effect of *Tfeb* silencing on glycolysis might also be mediated by its indirect effect on PKM2, which is phosphorylated at the Ser37 residue by ERK1/2 [27]. Dimeric p-Ser37 PKM2 is translocated into the nucleus, where it cooperates with transcription factors and phosphorylates histone H3, leading to the transcription of Cyclin D1 and glycolytic enzymes [24, 25, 27]. After *Tfeb* silencing, we found a reduction in the p-Ser37-PKM2 level and enzymatic activity. These results might reflect reduced glycolytic flux following the reduced uptake of glucose and the inhibition of ERK1/2 by the upregulation of DUSP-1, as discussed above. The rescue of ERK1/2 activity by DUSP-1 inhibition at least partially increased the PKM2 phosphorylation and Cyclin D1 expression levels.

Notably, it has been reported that DUSP-1 is involved in the control of energy expenditure, body mass, gluconeogenesis and insulin resistance onset by counteracting MAPK-dependent signals [80, 86, 87].

In cancer cells, the high degree of pyruvate conversion to lactate via aerobic glycolysis correlates with a reduction in the amount carbon entering the TCA cycle, resulting in the consumption of glutamine as a carbon source [88–93]. Different studies have suggested that glutamine is the major respiratory fuel for energy production in melanoma cells: it is consumed via classical glutaminolysis but is also the starting metabolite in citrate production, which is mediated through a reversal (reductive carboxylation) TCA cycle [11, 89, 90].

Moreover, when mitochondria become dysfunction, reductive carboxylation supports the proliferation of cancer cells [91–94]. In addition, a relationship among mitochondrial dysfunction, reductive carboxylation, and glycolysis has been described [91–94].

After *Tfeb* silencing, glycolysis levels, the glucose-fuelled TCA cycle rate, and mitochondrial activity were all reduced. Moreover, previous studies on TCA flux after [^14^C] glutamine addition suggested that glutamine is the starting metabolite in reverse TCA, which produced acetate, FAs and triglycerides. These phenomena were closely related to the alteration of cholesterol synthesis, as inferred by the rescue of cholesterol levels in *Tfeb* silenced cells, which partially reactivated glucose uptake and glycolysis and completely rescued OXPHOS and glutaminolysis with the subsequent decrease in FA and triglyceride quantity. A similar role for TFEB has been reported in regulating glutamine metabolism in pancreatic cancer [95]. However, the mechanism in melanoma and pancreatic cancer is likely different because in pancreatic cancer cells, direct control of TFEB on *Gls* transcription has been observed. These differences might be explained by other mechanisms, such as the effects exerted by enhancers and chromatin modifications in tissue-specific gene expression.

The effects of TFEB on melanoma metabolism are similar to the effects of MITF. MITF modulates the TCA cycle [96], FA saturation [97], the response to oxidative stress [98] and oxidative metabolism [70].

According to its involvement in autophagy flux control, TFEB may participate in many regulatory pathways of energy production and biosynthesis; in fact, it has been reported to regulate energy metabolism in cancer [28]. In particular, in different cell types, TFEB plays a crucial role in FA oxidation, OXPHOS, lipophagy and ketogenesis [47–49], mitochondrial biogenesis [29, 51, 52], the expression of glucose transporters and glycolytic enzymes, and the activation pathways related to glucose homeostasis [45].

For the first time, the present study demonstrates complex molecular signalling orchestrated by TFEB in the control of proliferation and metabolism in melanoma cells. Interestingly, the observation that a genetically induced reduction in TFEB level increased the antiproliferative effect exerted by a BRAF inhibitor suggests new possible therapeutic interventions targeting TFEB for managing melanoma and overcoming acquired resistance to standard therapies.

## Materials and methods

### Cells and genetic manipulation

The experiments were performed using (i) the D4M cell line obtained by backcrossing transgenic *Tyr::CreER;Braf*^*CA*^*;Pten*^*lox/lox*^ mice to C57BL/6 mice, resulting in Braf/Pten mice [57], and (ii) YUMM (Yale University Mouse Melanoma) cell lines obtained by backcrossing different transgenic mice carrying WT *Braf* (YUMM 4.1) or *Braf*V600E (YUMM 3.3, YUMM 1.7) to C57BL/6 mice [58].

The cells were tested for mycoplasma contamination using a Venor GeM Mycoplasma Detection kit (Thermo Fisher).

Endogenous *Tfeb* or *Dusp-1* was silenced in different melanoma cells by a specific sh-RNA lentivirus (sh-Tfeb or sh-Dusp-1 cells), and in all experiments, *Tfeb-* or *Dusp-1*-silenced cells were compared with control cells carrying scramble sh-RNA (scr-shRNA cells) (sh-Tfeb: TRCN0000085548, TRCN0000085549 TRCN0000085550, NM_011549; sh-Dusp-1: TRCN0000029020, TRCN0000376788, TRCN0000366485, TRCN0000366420, TRCN0000375399, NM_013642.3) cloned in a pLKO.1-puro nonmammalian vector. We verified the efficiency and specificity of the different shRNAs against TFEB or DUSP-1 and did not find any difference among them. Melanoma cells were transduced with specific lentiviral particles (MOI = 1) prepared according to the method described by Follenzi et al. [99]. The medium was replaced after 24 h, and cells stably expressing the lentivirus were selected with puromycin (1 μg/ml) for 24 h. We verified the expression of TFEB and DUSP-1 in melanoma cells by qPCR and immunoblot analyses. The TFEB-GFP-expressing construct was created by cloning the TFEB coding sequence (OriGene, cod. SC122773) in a pAcGFP-C1 vector (BD Bioscience). A TFEBS142A mutant was generated by inserting a single point mutation, while the TFEB ∆NLS mutant was created by deleting the sequence AGGAGACGAAGG, which corresponded to the protein sequence RRRR (245-248), via a Phusion site-directed mutagenesis kit (Thermo Fisher Scientific) as previously described [59, 61, 62]. Subconfluent cells were transfected with Lipofectamine (Thermo Fisher Scientific) according to the manufacturer’s protocol.

### Mouse allograft tumours

A total of 1 × 10^6^ ctrl, scr-shRNA and sh-Tfeb D4M cells with the corresponding lentiviral particles 3 days after transduction were resuspended in PBS and matrigel and then subcutaneously injected into the flank of immunocompetent C57BL/6 mice (at least 7 mice in each group). Tumour size was measured with a calliper, and tumour volume was calculated by the modified ellipsoid formula: length × (width)^2^/2. Allograft mice were maintained for 22 days.

### Tissue- and cell-staining analysis

For immunofluorescence staining, cells or tissue slices derived from tumours frozen in optimal cutting temperature (OCT) compound were washed in PBS, fixed in 4% paraformaldehyde (PFA), permeabilized in 0.1% Triton X-100 PBS for 5 min at 4 °C, saturated with 1% donkey serum in PBS (30 min), incubated with specific primary antibodies for 1 h at RT and then treated with appropriate Alexa Fluor-tagged secondary antibodies. (Thermo Fisher Scientific). Different fields of view (5–8) in each sample section were randomly chosen for analysis. When evaluating the same molecule in different samples, the laser power, gain and offset settings were maintained. The images were quantified using ImageJ software.

Immunofluorescence images were acquired with a TCS SPE confocal laser-scanning microscope (Leica Microsystems).

### Western blotting

Cells were washed twice with cold PBS, and proteins were extracted with a buffer containing 0.5 M Tris-HCl, pH 6.8; 5% sodium dodecyl sulfate (SDS); and 20% glycerol and quantified with the bicinchoninic acid (BCA) protein assay kit (Thermo Fisher Scientific). Equal amounts of each sample were separated by SDS‒PAGE (Bio–Rad) and transferred to PVDF membranes (Bio–Rad). Membranes were incubated with specific primary antibodies and HRP-conjugated secondary antibodies. Immunoreactive proteins were visualised with an enhanced chemiluminescence (ECL) system (Bio–Rad) acquired using a ChemiDoc Touch Gel Imaging System (Bio–Rad) and analysed with Image Lab software 5.2.1 (Bio–Rad).

The following antibodies were used: anti-TFEB (Bethyl – A303-673A), anti-Cyclin D1 (Abcam – ab16663), anti-CDK4 (Abcam – ab137675), anti-Rb (Abcam – ab181616), anti-pRb Ser807/811 (Cell Signaling Technology – cst#8516), anti-PCNA (Cell Signaling Technology – cst#2586), anti-pMEK1/2 Ser217/221 (Cell Signaling Technology – cst#9154), anti-MEK1/2 (Cell Signaling Technology – cst#9126), anti-pERK1/2 (Cell Signaling Technology - cst#9106), anti-ERK1/2 (Cell Signaling Technology – cst#9102), anti-GLS (Thermo Fisher Scientific – PA5-35365), anti-GS (Novus Biologicals – NB110-41404), anti-GLUT-1 (Abcam – ab652), anti-ASCT2 (V501) (Cell Signaling Technology – cst5345), anti-PKM2 (Invitrogen – PA5-23034), anti-pPKM2 Ser37 (Invitrogen – PA5-105500), anti-DUSP-1(E6T5S) (Cell Signaling Technology – cst35217), anti-SCAP (Invitrogen – PA5-115869), anti-SREBP2 (Invitrogen – PA1-338), anti-HMGCR (Invitrogen – PA5-95846) and anti-alpha-actin (Abcam - ab179467). Full and uncropped western blots are presented in Supplemental File.

### MSD technology

A MAP Kinase (Total Protein) Whole-Cell Lysate Kit and MAP Kinase Whole-Cell Lysate Kit MULTI-SPOT plates were used (Meso Scale Diagnostics, Rockville, MA, USA) to quantify total ERK1/2 and phospho-ERK1/2 protein levels according to the MSD manufacturer’s information. As indicated, the amount of phosphorylated protein was calculated using the following formula: % phosphoprotein = ((2 × phospho-signal)/(phospho-signal + total signal)) × 100.

### Gene expression

Total RNA was isolated from cells with a Maxwell RSC miRNA Tissue kit (Promega). The quality and concentration of the RNA were assessed with a NanoDrop ND-1000 spectrophotometer (Thermo Fisher Scientific). One microgram of the extracted RNA was converted to cDNA using a High-Capacity cDNA Reverse Transcription kit and random primers (Thermo Fisher Scientific).

Real-time PCR was performed using a CFX96 system (Bio–Rad) with TaqMan/Sybr PCR Universal Master Mix and specific TaqMan/Sybr assays. The experiments were performed in triplicate, and *Tbp* was used as the reference gene.

The following TaqMan assays were used: Tfeb (Mm00448968), Ccnd1 (Mm00432359), Cdk4 (Mm00726334), Pcna (Mm00448100), Dusp-1 (Mm00457272_g1), Scap (Mm01250176_m1) Srebf2 (Mm01306292_m1), Hmgcr (Mm01282499_m1) and Tbp (Mm01277042). To analyse the expression of genes involved in the specific pathways “protein phosphatases,” “carbohydrate metabolism” and “mitoenergy metabolism,” we used a PrimerPCR Pathway Plate purchased from Bio–Rad Laboratories.

### ChIP

ChIP was performed with a Zymo-Spin ChIP Kit (Zymo Research) according to the manufacturer’s instructions. Briefly, ~5 × 10^6^ crosslinked cells were sonicated for 6 cycles (30 s ON, 30 s OFF) at 4 °C using a Bioruptor Plus sonication device (Diagenode). After sonication, cell lysate was centrifuged at 12,000 × *g* for 10 min at 4 °C. The supernatant was diluted ninefold with chromatin dilution buffer and incubated with or without 5 µg of an anti-TFEB antibody (Bethyl) with rotation at 4 °C overnight. Then, ZymoMag Protein A beads were added for 1 h at 4 °C while rotating. Immunoprecipitated complexes were washed 3 times prior to elution. To reverse the crosslinking, the immunoprecipitate was incubated with Proteinase K at 65 °C for 90 min. Then, DNA was purified using Zymo-Spin IC columns. Enrichment of ChIP DNA was verified by qPCR with the primers and probes of specific sequences.

The primers and probe sequences used in this study were designed with the Integrated DNA Technologies PrimerQuest tool (IDT): Cdk4: (i) primer fw: AGATAAAGGGCCACCTCCA; primer rv: GATTATGGAAGGTGGCCCAAT probe: TTAGCCGAGCGTAAGGTGAGTGC; (ii) primer fw: CGCGGCCTGTGTCTATG; rv: GTAAGGTGAGTGCAGTCTCATC; probe: CAGATAAAGGGCCACCTCCAGAGC; and Dusp-1: (i) primer fw: CGCGGTGAAGCCAGATTA; rv: CCCGTTCTGCGGTTCTC; probe: CACAGGACACCGCACAAGATCGA; (ii) primer fw: GCCGATGACGTCTTTGCTT; rv: GGGAGAACGGTTTGTTTGTTTG; probe: CCGGTCACGTGTCTGCCATT.

### Luciferase reporter assay

Cells were seeded in 24-well plates at a density of 4 × 10^4^ cells per well. After ChIP-seq analysis [59] the relative TFEB MACS peak on the promoter gene was identified in D4M cells that were transfected with a pMCS-GreenRenillaLuc_Dusp-1_full promoter, pMCS-GreenRenillaLuc_Dusp-1_promoter_dell1 (Del1) (in which the putative TFEB-binding site had been deleted), or pMCS-GreenRenillaLuc_Dusp-1_promoter _dell2 (Del2) (in which 100 bp before and after the same putative TFEB-binding site had been deleted) synthesised with GeneArt support (Thermo Fisher Scientific) using Lipofectamine Reagent according to the manufacturer’s instructions.

Luciferase activity was analysed with a Pierce Renilla Luciferase Glow Assay Kit or the Dual-Luciferase^®^ Reporter (DLR™) Assay System using a GloMAX 20/20 luminometer (Turner Biosystems, Sunnyvale, CA, USA). The relative reporter activity was calculated by normalising the luciferase activity to the Renilla luciferase activity.

### Melanoma cell proliferation assay

Cells were plated into a 6-well plate and allowed to adhere overnight. The following day, the cells were treated with 0.5 µM and 1 µM PLX4720 purchased from Selleck Chemicals dissolved in dimethyl sulfoxide (DMSO) at a final concentration of 500 mM. Cells were allowed to grow for 72 h before being stained with 0.1% crystal violet (Sigma‒Aldrich) staining solution in 25% methanol. Absorbance was subsequently read at 592 nm with a plate reader. Relative growth was calculated as the absorbance read for cells treated with PLX4720 divided by the absorbance read for the untreated cells. Nonspecific absorbance was subtracted from both readings.

### Flow cytometry detection of cells in the S phase of the cell cycle

We quantified proliferation activity by measuring the proportion of cells that had incorporated EdU during the S phase of the cell cycle. Two hours after EdU incorporation (10 µM), cells were subjected to an aldehyde fixation and a detergent permeabilization protocol. EdU was detected after inducing a copper-catalysed covalent reaction between an azide group coupled to Alexa Fluor ® 488 dye and an alkyne present in the ethynyl EdU moiety. This process was achieved after a 30 min incubation at room temperature in the dark on a seesaw rocker with a cocktail of Click-iT ® EdU Flow Cytometry Assay Kit reagents (Invitrogen Corporation, Carlsbad, CA) following the manufacturer’s instructions. Finally, the cells stained with FxCycle™ Violet Stain (Invitrogen Corporation, Carlsbad, CA), diluted 1:500 in 1X Click-iT ® saponin-based buffer, was analysed to quantify the total DNA content. Sample cells were sorted using a low flow rate on a CyAn ADP LX nine-colour analyser (Beckman Coulter, Brea, CA). Data were analysed using Summit 4.3 software (Beckman Coulter, Brea, CA).

### Glycolysis

Cells were washed with fresh medium, resuspended at 1 × 10^5^ cells/ml in 0.2 ml of 100 mM TRIS 10 mM/EDTA I mM (pH 7.4), and sonicated on ice in two 10 s bursts. Tumour homogenates were resuspended in the same buffer and sonicated. Enzymatic activity in cell lysate was measured after incubation for 5 min at 37 °C. The protein content was measured using a BCA1 kit (Sigma, St. Louis, MO). The activity of PFK was measured spectrophotometrically following the procedure reported in [100]. ALDO activity was measured by using an Aldolase Activity Colorimetric Assay Kit (BioVision, Milpitas, CA). The activity of GAPDH, ENO and LDH was measured spectrophotometrically according to the procedure described in [101, 102]. For the GAPDH activity measurement, the cell lysate was incubated with 5 mM 3-phosphoglyceric acid, 1 U phosphoglycerate 3-kinase, 5 mM ATP and 2.5 mM NADH. For the ENO activity measurement, the cell lysate was incubated with 10 mM MgCl_2_, 100 mM KCl, 1 mM 2-phosphoglyceric acid, 0.4 mM ADP, 6.8 U/mL Pk, 9.9 U/mL Ldh, and 0.2 mM NADH. The PK activity was measured with a pyruvate kinase assay kit (Abcam, Cambridge, UK). For all assays of glycolytic enzymes, the activity levels were monitored by measuring the absorbance variation at 340 nm with a Synergy HTX 96-well microplate reader (Bio-Tek Instruments).

### Lactate quantification

Lactate production was measured with a lactate assay kit (Sigma) following the manufacturer’s protocol, and the signals were measured with a Synergy HTX 96-well microplate reader (Bio-Tek Instruments).

### Glutamine catabolism and synthesis

Cells were washed with PBS, detached from the wells by gentle scraping, centrifuged at 13,000 × *g* for 5 min at 4 °C, resuspended in 250 µL of buffer A (150 mmol/L KH_2_PO_4_, 63 mmol/L Tris/HCl, and 0.25 mmol/L EDTA; pH 8.6) and sonicated. Whole-cell lysates were incubated for 30 min at 37 °C in a quartz cuvette in the presence of 50 µL of 20 mmol/L L-glutamine and 850 µL of buffer B (80 mmol/L Tris/HCl, 20 mmol/L NAD^+^, 20 mmol/L ADP, and 3% v/v H_2_O_2_; pH 9.4). The absorbance of NADH was monitored at 340 nm using a Lambda 3 spectrophotometer (PerkinElmer). The kinetics were linear throughout the assay. The results are expressed as µmol NADH/min/mg cell protein and are considered to be indexes of the activity of GLS plus GLU DH. GLS activity was calculated by subtracting the rate obtained with the second assay from the rate obtained with the first assay. The enzymatic activity of GS was measured spectrophotometrically using a glutamine synthetase microplate assay kit (Cohesion Biosciences Ltd., London, UK). The alpha-ketoglutarate concentration was determined via coupled enzyme assay (Sigma; catalogue #: MAK054), which yields a colorimetric (570 nm) product in an amount proportional to the amount of alpha-ketoglutarate in the sampled cells.

### Glutamine/glutamate uptake and metabolism

To measure glutamine uptake and metabolism, 1 × 10^6^ cells were labelled with 1 μCi [^14^C]-L-glutamine (PerkinElmer) for 30 min, rinsed with ice-cold PBS and sonicated. An aliquot was used to quantify intracellular proteins. [^14^C]-L-glutamate and [^14^C]-L-glutamine within cell lysates were separated via ion exchange chromatography. The radioactivity of the eluate containing [^14^C]-L-glutamate and [^14^C]-L-glutamine was measured with a liquid scintillation counter and is expressed as μmol/mg cellular proteins. The ratio of [^14^C]-L-glutamate/[^14^C]-L-glutamine was considered an index of glutamine consumption. In the case of glutamate uptake, 1 × 10^6^ cells were labelled with 1 μCi [^14^C]-L-glutamate (PerkinElmer). The cells were processed as described above, and the intracellular amount of [^14^C]-L-glutamate was measured with a liquid scintillation counter.

### TCA flux

Glucose flux through the TCA cycle was measured by radiolabelling 1 × 10^6^ cells with 2 μCi [6-^14^C]-glucose (55 mCi/mmol; PerkinElmer, Waltham, MA, USA), [1-^14^C]-acetic acid (1 mCi/ml, PerkinElmer) or [^14^C]-L-glutamine (200 mCi/mmol, PerkinElmer). Cell suspensions were incubated for 1 h in a closed experimental system to trap the ^14^CO_2_ released from [^14^C]-glucose, [^14^C]-acetic acid], and [^14^C]-L-glutamine. The reaction was stopped by injecting 0.5 ml of 0.8 N HClO_4_. The amount of glucose transformed into CO_2_ through the TCA cycle was calculated as described in [103].

The enzymatic activity of citrate synthase, aconitase, IDH, alpha-ketoglutarate dehydrogenase, and SDH were measured on the basis of 10 µg of mitochondrial proteins using a citrate synthase assay kit (Sigma), an aconitase assay kit (Cayman Chemical, Ann Arbor, MI), an isocitrate dehydrogenase activity assay kit (Sigma), an alpha-ketoglutarate assay kit (Abcam), and a succinate dehydrogenase activity colorimetric assay kit (BioVision), as per the respective manufacturer’s instructions.

### Mitochondrial extraction and electron transport chain (ETC)

To extract mitochondria, cells or tumours were lysed in mitochondria lysis buffer (50 mM Tris-HCl; 100 mM KCl; 5 mM MgCl_2_; 1.8 mM ATP; and 1 mM EDTA, pH 7.2) supplemented with Protease Inhibitor Cocktail III (Sigma), 1 mM phenylmethylsulfonyl fluoride (PMSF) and 250 mM NaF. Samples were clarified by centrifugation. Supernatants, corresponding to the cytosolic fraction, were used for cytosolic reactive oxygen species (ROS) measurements. Pellets containing mitochondria were washed once with lysis buffer and resuspended in mitochondrion resuspension buffer (250 mM sucrose, 15 mM K_2_HPO_4_, 2 mM MgCl_2_, and 0.5 mM EDTA). Samples were sonicated and used for the measurement of the protein content with a BCA protein assay kit (Sigma) and for metabolic assays. To measure complex I activity, unsonicated mitochondrial samples were resuspended in 0.2 ml of buffer 1 A (5 mM KH_2_PO_4_, 5 mM MgCl_2_, and 5% w/v BSA), incubated at room temperature and then treated with 0.1 ml of buffer 1B (25% w/v saponin, 50 mM KH_2_PO_4_, 5 mM MgCl_2_, 5% w/v BSA, and 0.12 mM oxidised ubiquinone, which is shuttle that moves complex I to complex III; 2.5 mM antimycin A, which inhibits complex III; and 0.2 mM NaN_3_, which blocks complex IV; pH 7.5). NADH (1.5 mM) was added to the mix as an electron donor. The rate of NADH oxidation was measured for 5 min at 37 °C, and the absorbance was read at 340 nm.

Complex II activity was measured to represent the rate of the electron transfer between complex II and complex III. Unsonicated mitochondrial samples were resuspended in 0.1 ml of buffer 2 A (50 mM KH_2_PO_4_, 7.5 mM MgCl_2_, 25% w/v saponin, and 20 mM succinic acid; pH 7.2). Buffer 2B (50 mM KH_2_PO_4_, 7.5 mM MgCl_2_, 5% w/v BSA, and 30 mM succinic acid, as a substrate of complex II; 0.12 mM oxidised ubiquinone, as a shuttle to move electrons from complex II to complex III; 0.12 mM oxidised cytochrome c, as an acceptor of electrons flowing from complex II to complex III; 5 mM rotenone to prevent electron flux from complex I; and 0.2 mM NaN_3_ to block complex IV) was added. The rate of the reduction of cytochrome c was measured for 5 min at 37 °C, with the absorbance read at 550 nm.

The activity of complex III was measured in the same samples to represent the electron flux from complex I to complex III. One minute after the addition of NADH, an inducer of electron flow, 5 mM rotenone, which blocks the activity of complex I, was added. The rate of the reduction of cytochrome c, which is dependent on the activity of complex III only in the presence of rotenone, was measured for 5 min at 37 °C, and the absorbance was read at 550 nm.

To measure the activity of complex IV, the oxidation rate of cytochrome c (in reduced form generated by complex III) was measured. Twenty micrograms of unsonicated mitochondrial samples were resuspended in 0.1 ml of buffer 4 A (50 mM KH_2_PO_4_, 20 mM succinic acid, and 25% w/v saponin; pH 7.2) and incubated for 30 min at room temperature. Then, 0.2 ml of buffer 4B (50 mM KH_2_PO_4_; 5 mM rotenone, which prevents electrons from moving from complex I to complex III; 30 mM succinic acid, as a substrate of complex II and an electron generator; and 0.03 mM reduced cytochrome c as an acceptor of electrons flowing from complex III to complex IV) was added. The oxidation rate of cytochrome c was measured for 5 min at 37 °C, and the absorbance was read at 550 nm.

### Mitochondrial ATP quantification

The ATP levels in mitochondrial extracts were measured with an ATP bioluminescence assay kit (Sigma‒Aldrich, St. Louis, MO USA). ATP was quantified in relative light units (RLU), which were converted into nmoles ATP/mg mitochondrial proteins, according to a previously established calibration curve.

### Total cellular ATP quantification

The intracellular ATP level was measured using a Cell Titer-Glo Luminescent Cell Viability Assay kit (Promega) following the manufacturer’s instructions. The results are expressed as RLU.

### mPTP opening

The opening of the mPTP, considered an index of damaged mitochondria, was measured using a Mitochondrial Permeability Transition Pore Assay Kit (BioVision) according to the manufacturer’s instructions. The intracellular fluorescence was measured at an excitation λ wavelength of 488 nm on a Synergy HTX 96-well microplate reader (Bio-Tek Instruments). The results are expressed as relative fluorescence units (RFU)/mg cellular proteins.

### Mitochondrial Tbars

The extent of oxidative damage was measured in mitochondrial extracts by using a Lipid Peroxidation (4-HNE) Assay Kit (Abcam), which is used to evaluate 4-hydroxy-nonenale, a thiobarbituric reactive substance that is an index of lipid peroxidation. The results are expressed as nmoles/mg mitochondrial proteins.

### SOD activity

Mitochondrial separation was performed, and the activity of SOD2 was measured in the mitochondrial extracts. Ten micrograms of protein from the extract was incubated with 50 μmol/L xanthine, 5 U/mL xanthine oxidase, and 1 μg/mL oxidised cytochrome c at 37 °C. The cytochrome c reduction rate, which was inhibited by SOD, was monitored for 5 min by reading the absorbance at 550 nm with a Lambda 3 spectrophotometer (PerkinElmer). The results are expressed as μmoles of reduced cytochrome c/min/mg of mitochondrial proteins.

### Cholesterol and isoprenoid synthesis

The de novo synthesis of cholesterol, FPP, GGP and ubiquinone was measured by radiolabelling 1 × 10^6^ cells (after overnight starvation) with 1 µCi [^3^H]acetate (3600 mCi/mmol; Amersham Bioscience, Piscataway, NJ) for 24 h. The cells were washed twice with PBS and scraped off the substrate and added in 200 µl of PBS. Methanol (0.5 ml) and hexane (1 ml) were added to the cell suspension, which was stirred at room temperature for 1 h and centrifuged at 2000 × *g* for 5 min. The upper phase containing hexane was transferred to a new test tube, and the lower phase was supplemented with 1 ml of hexane and stirred overnight. After a 5 min centrifugation at 2000 × *g*, the upper phase was added to the previously obtained hexane phase, and the solvent was allowed to evaporate at room temperature for 24 h. Cellular lipid extracts obtained via this separation were resuspended in 30 µl of chloroform and then subjected to thin layer chromatography (TLC) using a 1:1 (v/v) ether/hexane solution as the mobile phase. Each sample was spotted on precoated LK6D Whatman silica gels (Merck, Darmstadt, Germany) and allowed to run for 30 min. Solutions of 10 µg/ml cholesterol, GGPP and ubiquinone were used as the standards. The plates were exposed for 1 h to an iodine-saturated atmosphere, and the migrated spots were cut out of the gel. The radioactivity in the excised spots was measured by liquid scintillation counting using a Tri-Carb Liquid Scintillation Analyser (PerkinElmer, Waltham, MA). Cholesterol, GGPP and ubiquinone synthesis is expressed as pmoles/10^6^ cells, according to the titration curves previously established.

A fluorometric Cholesterol/Cholesteryl Ester Assay Kit—Quantitation was used to measure the level of free cholesterol in cell lysates in accordance with the manufacturer’s instructions. The results are expressed as mg of cholesterol or cholesteryl esters/mg of cellular proteins.

### Statistical analysis

The sample sizes were not selected and were consistent with those used in similar experiments performed in other laboratories investigating melanoma development. No statistical methods were used to predetermine the sample size, and we did not randomise the samples because our experimental design did not require this strategy. The investigators were not blinded to the allocation of the samples during the experiments or analyses. The data are presented as the means ± SEMs.

Statistical analyses were performed using Excel (Microsoft) and Prism (GraphPad) software. Appropriate statistical tests were performed as indicated in the Results section, and *p* < 0.05 was considered to indicate statistical significance in all the experiments.

## Supplementary information

SUPPLEMENTAL FIGURE LEGENDS

SUPPLEMENTAL FIG S1

SUPPLEMENTAL FIG S2

SUPPLEMENTAL FIG S3

SUPPLEMENTAL FIG S4

SUPPLEMENTAL FIG S5

SUPPLEMENTAL FIG S6

SUPPLEMENTAL FIG S7

SUPPLEMENTAL FIG S8

FIG WESTERN BLOT

Author check list

## Data Availability

All data generated during this study are available from the corresponding author on reasonable request.
