# Supplementary material for: TFEB inhibition induces melanoma shut-down by blocking the cell cycle and rewiring metabolism
Source: Cell Death Dis. 2023 May 9;14(5):314. doi: 10.1038/s41419-023-05828-7 (PMC10170071; doi:10.1038/s41419-023-05828-7)
Supplement: Supplementary file 1 — SUPPLEMENTAL FIGURE LEGENDS [file 41419_2023_5828_MOESM1_ESM.docx]

**SUPPLEMENTARY FIGURE LEGENDS**

**Supplementary figure 1. Melanoma cell characterization.**

1. Representative western blots (n=3) show the amounts of TFEB, p-MEK1/2, MEK, p-ERK1/2, ERK1/2 and alpha-actin protein in D4M, YUMM 4.1, YUMM 3.3 and YUMM 1.7 melanoma cells.
2. qPCR measurement of the *Tfeb* expression level in scr-shRNA D4M cells and D4M infected with 3 different specific lentiviral Tfeb sh-RNAs: sh-Tfeb #1, sh-Tfeb #2 and sh-Tfeb #3 D4M. The data are expressed as the relative fold changes in sh-Tfeb D4M cells compared with the expression in scr-shRNA D4M cells after normalization to the level of the housekeeping gene *Tbp* (n=3 independent experiments; values are reported as the means±SEMs; ***p<0.0001, **p<0.001 for sh-Tfeb versus scr-shRNA D4M cells, as determined by Student’s *t* test).
3. qPCR measurement of *Tfeb* expression in scr-shRNA and sh-Tfeb YUMM 4.1, 3.3 and 1.7 cells. The data are expressed as the relative fold changes in sh-Tfeb cells compared with the expression in scr-shRNA cells after normalization to the level of the housekeeping gene *Tbp* (n=3 independent experiments; values are reported as the means±SEMs; **p<0.001 for sh-Tfeb versus scr-shRNA cells, as determined by Student’s *t* test).
4. Representative western blots (n=3) show the levels of TFEB, p-ERK1/2, ERK1/2, DUSP-1 and alpha-actin protein in D4M, YUMM 4.1, YUMM 3.3 and YUMM 1.7 melanoma cells.
5. qPCR measurements and representative western blots showing the *Tfe3*, *Tfec*, and *Mitf* expression levels in scr-shRNA and sh-Tfeb D4M cells. qPCR data are expressed as the relative fold changes in sh-Tfeb cells compared with the expression in scr-shRNA cells after normalization to the level of the housekeeping gene *Tbp* (n=3 independent experiments; values are reported as the means±SEMs; p=ns for sh-Tfeb versus scr-shRNA cells, as determined by Student’s *t* test).

**Supplementary figure 2. Effects of *Tfeb* silencing on Dusp-1 transcription.**

1. Heatmap showing the expression levels of general phosphatase genes in sh-Tfeb compared to scr-shRNA D4M cells. The data are expressed as the relative fold-change of sh-Tfeb D4M cells compared with the expression in scr-shRNA cells after normalization to the level of the housekeeping gene *Tbp*. (Red: upregulated genes).
2. qPCR of Dusp-1 expression in scr-shRNA and sh-Tfeb D4M and YUMM cells. The data are expressed as the relative fold change in sh-Tfeb cells compared with the expression in scr-shRNA cells after normalization to the level of the housekeeping gene *Tbp* (n=3 independent experiments, values as the means±SEMs; ***p<0.0001, **p<0.001 and *p<0.01 sh-Tfeb versus scr-shRNA cells by Student’s *t* test).
3. Representative snapshot shows TFEB binding to *Dusp-1* promoter.
4. Confocal microscopy analysis of TFEB and actin expression in scr-shRNA and sh-Tfeb D4M after addiction of vector, TFEBS142A, TFEB ∆NLS and WT TFEB and incubation with anti-TFEB and phalloidin-647 antibody (scale bar: 25 µm). The bar graphs show the quantification of % of cells with TFEB nuclear positivity (n=15 images per condition pooled from three different tumours; values as means±SEMs; ***p<0.0001 for all condition vs scr-shRNA D4M as determined by Student’s *t*-test).

**Supplementary figure 3. Effects of *Tfeb* silencing on Dusp-1 expression, and the role of TFEB in melanoma cell proliferation.**

1. Analysis of TFEB modulation of the *Dusp-1* and *Cdk4* promoters in scr-shRNA, sh-Tfeb and sh-Tfeb+TFEBS142A D4M cells. The bar graph shows the relative percentage of luciferase activity in the cells after transfection of the *Dusp-1 or Cdk4* full-length promoter, promoter deletion 1 (Del1) and promoter deletion 2 (Del2) (n=3 independent experiments; the means±SEMs; ***p<0.0001, **p<0.001 and *p<0.01 for all conditions versus scr-shRNA D4M cells; ^###^p<0.0001 and ^#^p<0.01 for all conditions + promoter deletion mutant versus all conditions + full promoter; °°°p<0.0001 for sh-Tfeb + TFEBS142A + full promoter D4M cells versus sh-Tfeb + full promoter D4M cells as determined by Student’s *t* test).
2. Flow cytometry evaluation of scr-shRNA and sh-Tfeb YUMM 4.1, 3.3 and 1.7 cell proliferation by measuring cell EdU incorporation during the S phase of the cell cycle. Total DNA content was measured with FxCycle™ Violet Stain. The relative percentage of cells in +S phase is indicated.
3. qPCR measurement of *Cdk4*, *Ccnd1*, and *Pcna* expression in scr-shRNA and sh-Tfeb YUMM 4.1, 3.3 and 1.7 cells. The data are expressed as the relative fold change in sh-Tfeb YUMM cells compared with the expression in scr-shRNA YUMM cells after normalization to the level of the housekeeping gene *Tbp* (n=3 independent experiments, values are reported as the mean±SEM; ***p<0.0001, **p<0.001 and *p<0.01 sh-Tfeb versus scr-shRNA cells, as determined by Student’s *t* test).
4. Representative snapshot shows TFEB binding to the *Ccnd1* promoter.

**Supplementary figure 4. Effects of *Dusp-1* silencing in sh-Tfeb D4M cells.**

A) qPCR measurement of *Dusp-1* expression in scr-shRNA (#1-5) D4M cells infected with 5 different specific lentiviral *Dusp-1* sh-RNAs. The data are expressed as the relative fold changes in sh-Dusp1 D4M cells compared with the expression in scr-shRNA D4M cells after normalization to the level of the housekeeping gene *Tbp* (n=3 independent experiments; values are reported as the means±SEMs; ***p<0.0001 and **p<0.001 for sh-Dusp1 versus scr-shRNA D4M cells, as determined by Student’s *t* test).

B) qPCR of *Dusp-1* expression in scr-shRNA, sh-Tfeb, sh-Dusp-1 or sh-Tfeb+sh-Dusp-1 D4M cells. The data are expressed as the relative fold changes in all gene-expressing D4M cells compared with the expression in the scr-shRNA D4M cells after normalization to the level of the housekeeping gene *Tbp* (n=3 independent experiments; values are reported as the means±SEMs; ***p<0.0001 and *p<0.01 for all D4M cells versus scr-shRNA D4M cells, as determined by Student’s *t* test).

C) Flow cytometry evaluation of scr-shRNA, sh-Tfeb, sh-Dusp-1 or sh-Tfeb+sh-Dusp-1 D4M cell proliferation by measuring cell EdU incorporation during the S phase of the cell cycle. Total DNA content was measured with FxCycle™ Violet Stain. The relative percentage of cells in the S phase is indicated.

D) Representative western blots show the phosphorylated- and total PKM2 expression levels in scr-shRNA and sh-Tfeb D4M cells with or without BCI (1 µM, 30’-1 h) treatment. The bar graph shows the densitometry results expressed as the ratio of phosphorylated protein to- total protein (n=3 independent experiments; the means±SEMs; ***p<0.0001, **p<0.001 and *p<0.01 for all samples versus scr-shRNA D4M cells, ^#^p<0.01 for sh-Tfeb + BCI-treated versus sh-Tfeb D4M cells, as determined by Student’s *t* test).

**Supplementary figure 5. Metabolic regulation of melanoma cell activity after *Tfeb* silencing.**

1. qPCR measurement of *Pfkm, Aldo, Gapdh, Eno, Pkm2, Ldh, Cs, Idh*, *Sdh* and *Mdh* expression levels in scr-shRNA and sh-Tfeb D4M cells. The data are expressed as the relative fold changes in sh-Tfeb D4M cells compared with the expression in scr-shRNA D4M cells after normalization to the level of the housekeeping gene *Tbp* (n=3 independent experiments; values are reported as the means±SEMs; p=ns for sh-Tfeb versus scr-shRNA D4M cells, as determined by Student’s t test).

B-E-G) Representative western blots show the GLUT-1, SLC1A5, GLS, GS and alpha-actin expression levels in scr-shRNA and sh-Tfeb D4M cells.

C) The bar graph shows the levels of acetate in scr-shRNA and sh-Tfeb D4M cells (n=3 independent experiments; values are reported as the means±SEMs; **p<0.001 for sh-Tfeb versus scr-shRNA D4M cells, as determined by Student’s *t* test).

D, F) The bar graphs show the levels of GLS, GLU DH and GS activity in scr-shRNA and sh-Tfeb D4M cells (n=3 independent experiments; values are reported as the means±SEMs; **p<0.001 for sh-Tfeb versus scr-shRNA D4M cells, as determined by Student’s *t* test).

H) qPCR measurement of *Scap, Srebf2* and *Hmgcr* expression levels in scr-shRNA and sh-Tfeb D4M cells. The data are expressed as the relative fold changes in sh-Tfeb D4M cells compared with the expression in scr-shRNA D4M after normalization to the level of the housekeeping gene *Tbp* (n=3 independent experiments; values are the means±SEMs; ***p<0.0001 and **p<0.001 for sh-Tfeb versus scr-shRNA D4M cells, as determined by Student’s *t* test).

**Supplementary figure 6. Combinatory effects of PLX4720 and *Tfeb* silencing.**

1. Representative images and quantification of cell growth of scr-shRNA and sh-Tfeb D4M cells after treatment with 0.5 µM and 1 µM PLX4720 for 48 h. (n=3 independent experiments; values are the means±SEMs; ***p<0.0001 for all conditions versus scr-shRNA D4M, ^##^p<0.001 for sh-Tfeb+PLX4720 versus sh-Tfeb D4M cells as determined by Student’s *t* test).
2. qPCR of *Tfeb, Ccnd1* and *Pcna* expression in scr-shRNA and sh-Tfeb D4M cells treated or not treated with PLX4720 (1 µM, 24 h). The data are expressed as the relative fold changes in sh-Tfeb D4M cells compared with the expression in scr-shRNA D4M cells after normalization to the level of the housekeeping gene *Tbp* (n=3 independent experiments; values are reported the means±SEMs; ***p<0.0001 or **p<0.001 for all conditions versus scr-shRNA D4M cells, ^#^p<0.01 for sh-Tfeb +PLX4720 treated versus sh-Tfeb D4M cells as determined by Student’s *t* test).
3. Representative western blots show the TFEB, p-ERK1/2, ERK-1/2, Cyclin D1, PCNA and alpha-actin expression levels in scr-shRNA and sh-Tfeb D4M treated with or without PLX4720 (1 µM, 24 h).
4. Flow cytometry evaluation of scr-shRNA and sh-Tfeb D4M cell proliferation with or without PLX4720 treatment (1 µM, 24 h) by measuring cell EdU incorporation during the S phase of the cell cycle. Total DNA content was measured with FxCycle™ Violet Stain. The relative percentage of cells in S phase is indicated.
5. The bar graphs show the level of total cellular ATP in scr-shRNA and sh-Tfeb D4M cells with or without PLX4720 treatment (1 µM, 48 h) (n=3 independent experiments; values are reported as the means±SEMs; ***p<0.0001 for all conditions versus scr-shRNA- D4M cells,^###^p<0.0001 for sh-Tfeb +PLX4720-treated versus sh-Tfeb D4M cells as determined by Student’s *t* test).

**Supplementary figure 7. TFEB expression in melanoma tumours.**

Confocal microscopy analysis of TFEB expression in Ctrl, scr-shRNA and sh-Tfeb D4M tumours after incubation with an anti-TFEB antibody (scale bar: 25 µm). The bar graphs show the TFEB mean intensity/tumour area (n=15 images per condition pooled from three different tumours; values are reported the means±SEMs; ***p<0.0001 for sh-Tfeb versus ctrl D4M tumours, and ^###^p<0.0001 for sh-Tfeb versus sh-RNA D4M tumours as determined by Student’s *t* test).

**Supplementary figure 8. *Tfeb* silencing inhibits glucose and glutamine metabolism, the TCA cycle, mitochondrial activity and cholesterol synthesis in melanoma tumours.**

1. The bar graph shows the activity of enzymes involved in glucose metabolism in Ctrl, scr-shRNA and sh-Tfeb D4M tumours (the activity of PFK, ALDO, ENO, PK, and LDH was measured as nmol NAD^+^/min/mg protein; the activity of GAPDH was measured as nmol NADH/min/mg protein) (n=5 ctrl tumours, n=9 scr-shRNA and sh-Tfeb D4M tumours; values are reported as the means±SEMs; ***p<0.0001 for sh-Tfeb versus scr-shRNA D4M tumours, as determined by Student’s *t* test).
2. The bar graph shows the activity of enzymes involved in the TCA cycle in Ctrl, scr-shRNA and sh-Tfeb D4M tumours (the activity of IDH and alpha-KGDH was measured as nmol NADH/min/mg protein; the activity of CS and ACO was measured as nmol citrate or isocitrate/min/mg protein; and the activity of SDH was measured as nmol FADH2/min/mg protein) (n=5 ctrl tumours, n=9 scr-shRNA and sh-Tfeb D4M tumours; values are reported as the means±SEMs; ***p<0.0001 for sh-Tfeb versus scr-shRNA D4M tumours, as determined by Student’s *t* test).

C, D) The bar graphs show the activity of GLS and GS in Ctrl, scr-shRNA and sh-Tfeb D4M tumours (n=5 ctrl tumours, n=9 scr-shRNA and sh-Tfeb D4M tumours; values are reported as the means±SEMs; ***p<0.0001 for sh-Tfeb versus scr-shRNA D4M tumours, as determined by Student’s *t* test).

E) The bar graph shows the rate of mitochondrial ATP synthesis in Ctrl, scr-shRNA and sh-Tfeb D4M tumours (n=5 ctrl tumours, n=9 scr-shRNA and sh-Tfeb D4M tumours; values are reported as the means±SEMs; ***p<0.0001 for sh-Tfeb versus scr-shRNA D4M tumours, as determined by Student’s *t* test).

F) The bar graph shows the levels of cholesterol in Ctrl, scr-shRNA and sh-Tfeb D4M tumours (n=5 ctrl tumours, n=9 scr-shRNA and sh-Tfeb D4M tumours; values are reported as the means±SEMs; ***p<0.0001 for sh-Tfeb versus scr-shRNA D4M tumours, as determined by Student’s *t* test).
