# Supplementary figures and images for: TFEB inhibition induces melanoma shut-down by blocking the cell cycle and rewiring metabolism
Source: Cell Death Dis. 2023 May 9;14(5):314. doi: 10.1038/s41419-023-05828-7 (PMC10170071; doi:10.1038/s41419-023-05828-7)

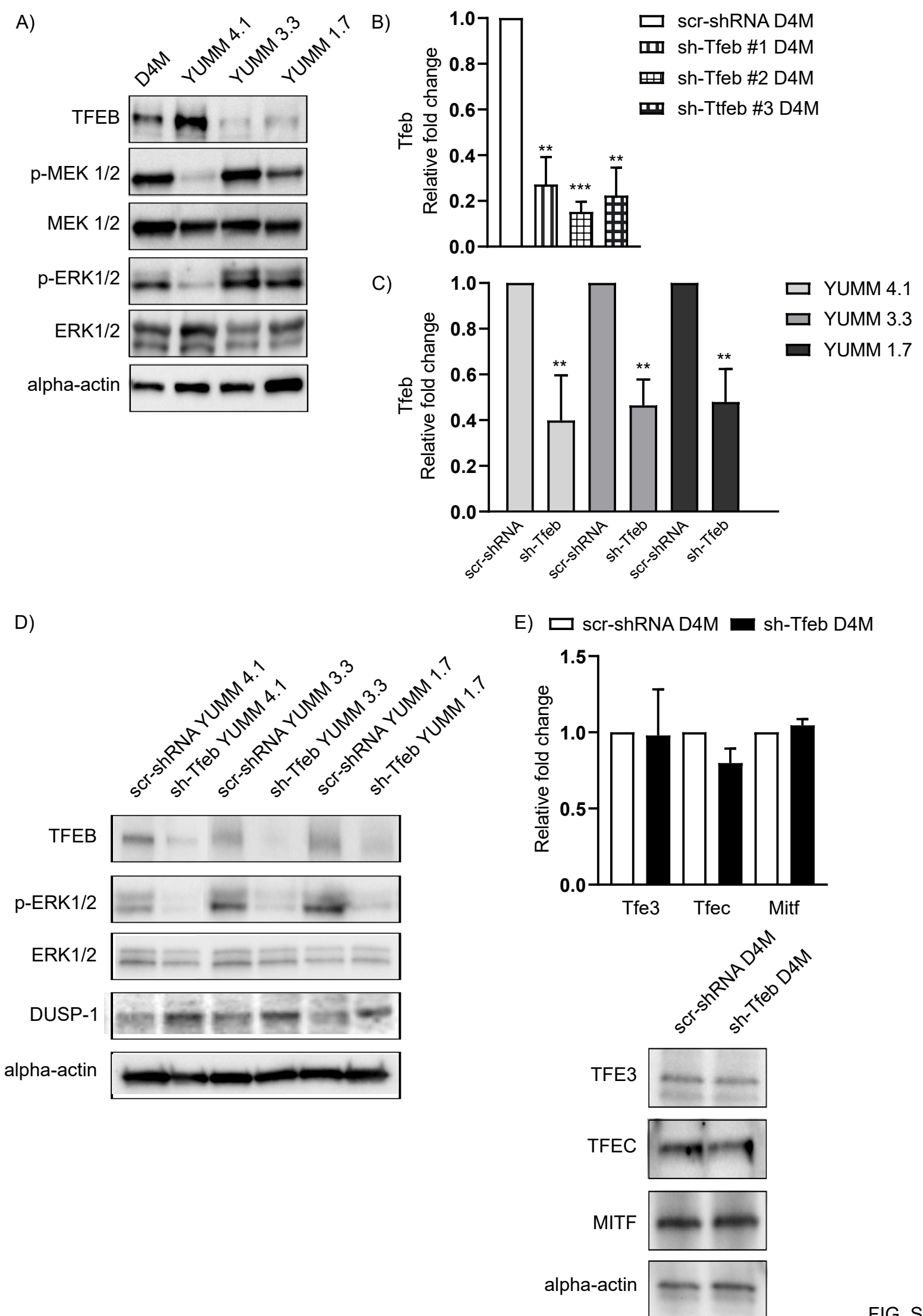

FIG. S1

Supplement: Supplementary file 2 — SUPPLEMENTAL FIG S1 [file 41419_2023_5828_MOESM2_ESM.pdf]

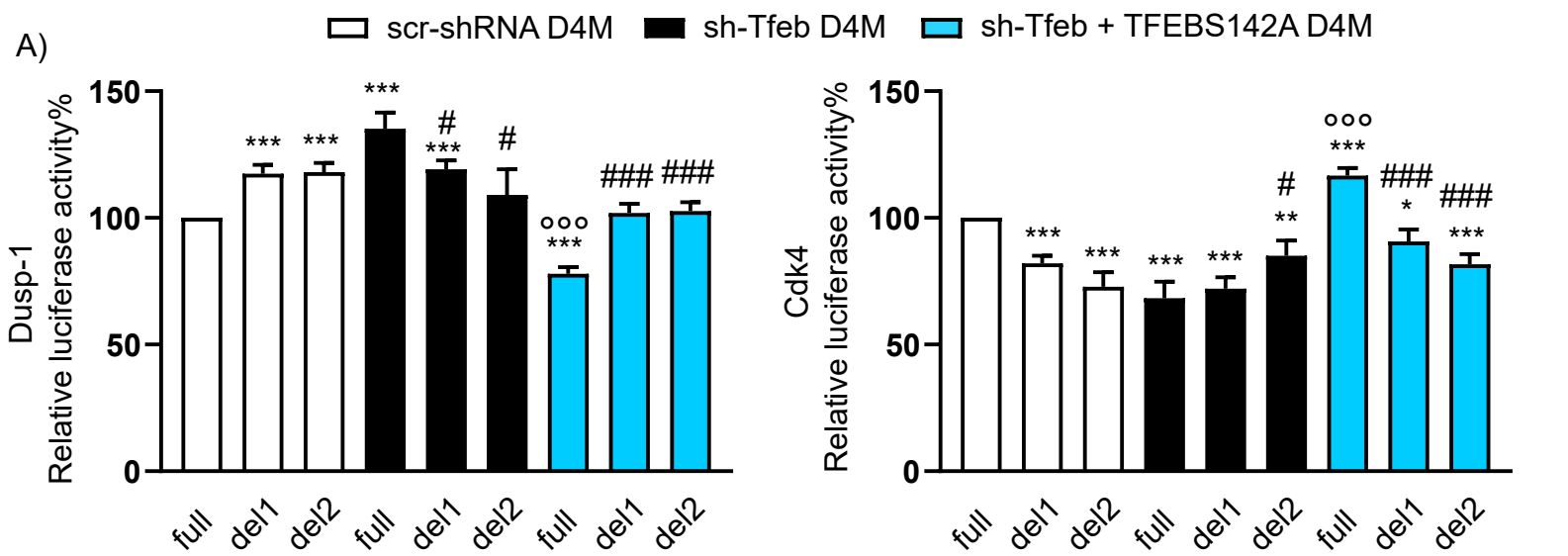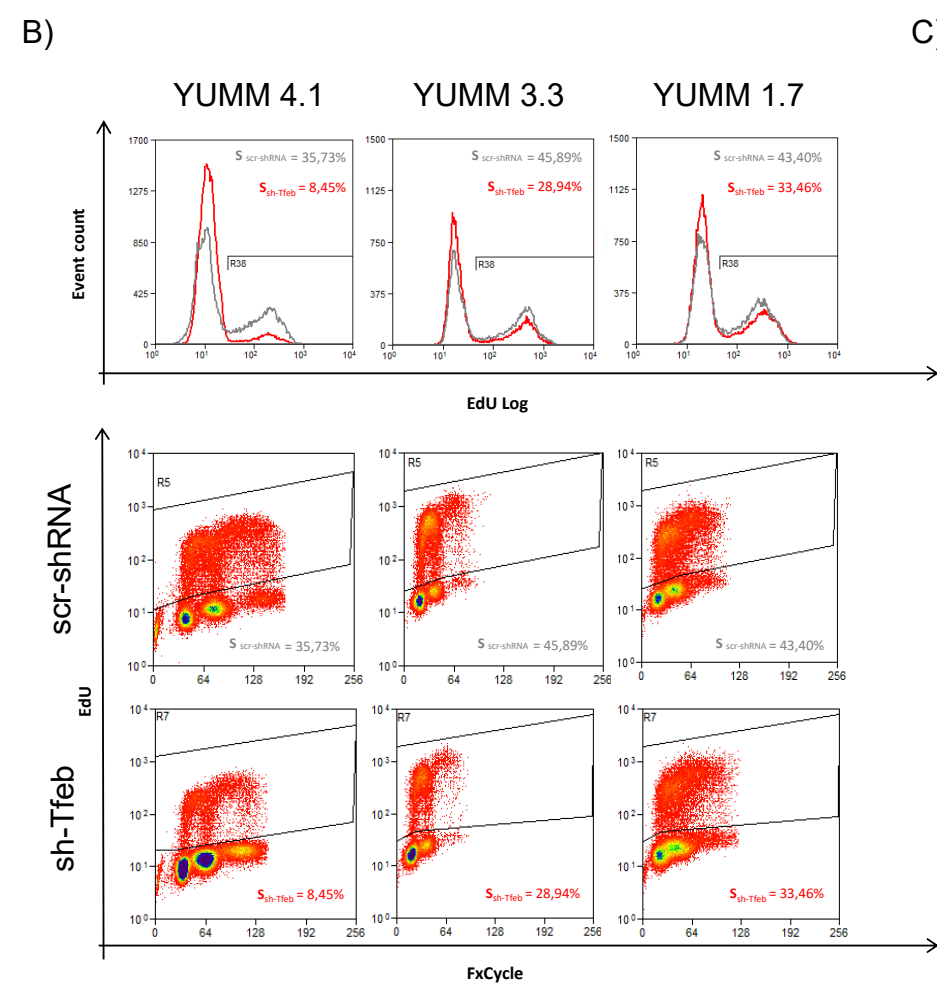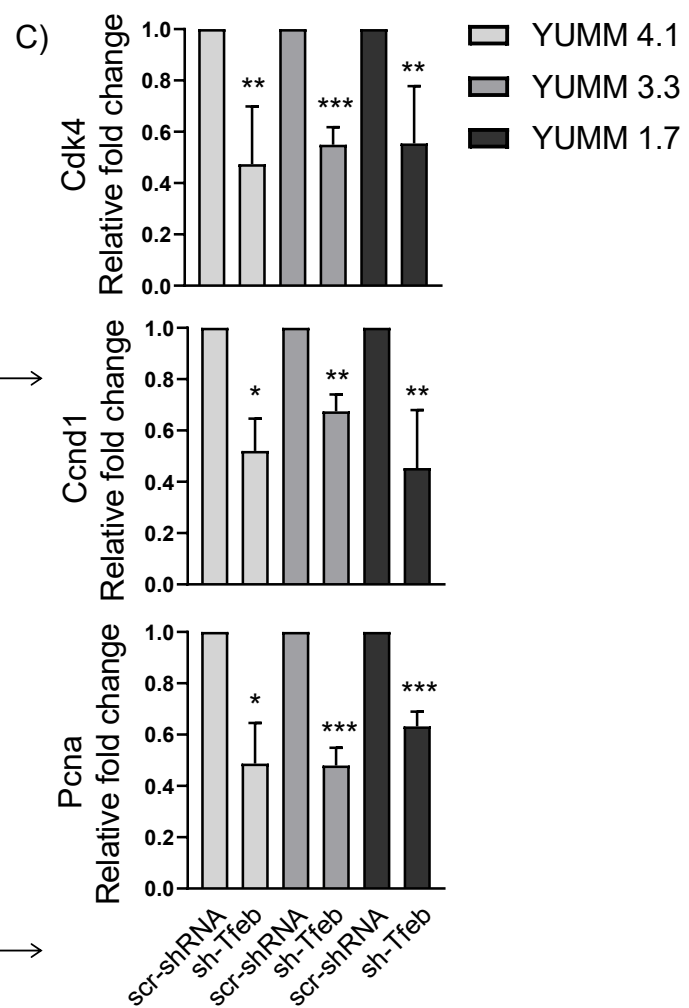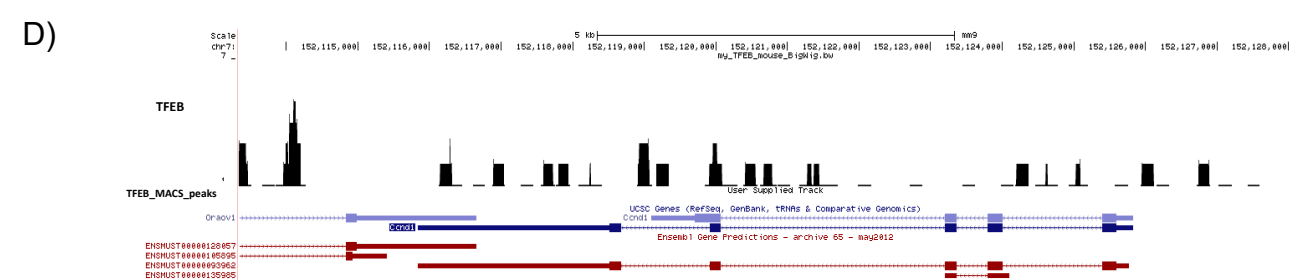

FIG S3

Supplement: Supplementary file 4 — SUPPLEMENTAL FIG S3 [file 41419_2023_5828_MOESM4_ESM.pdf]

A)

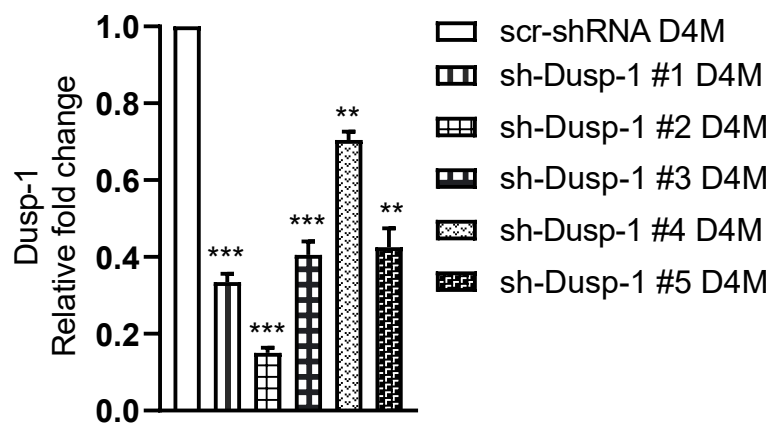

B)

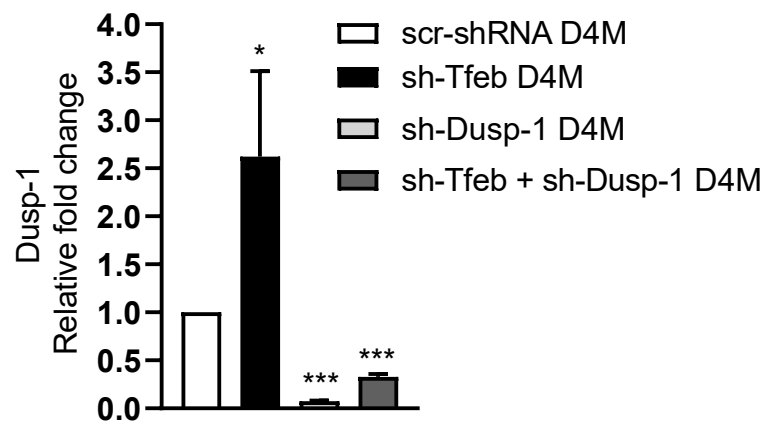

C)

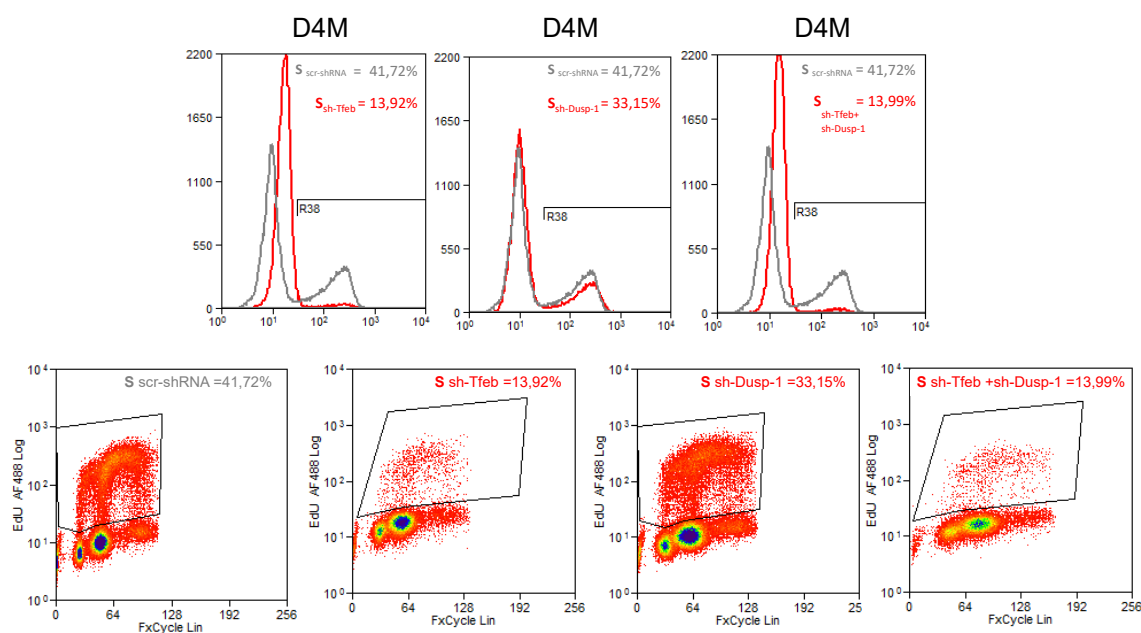

D)

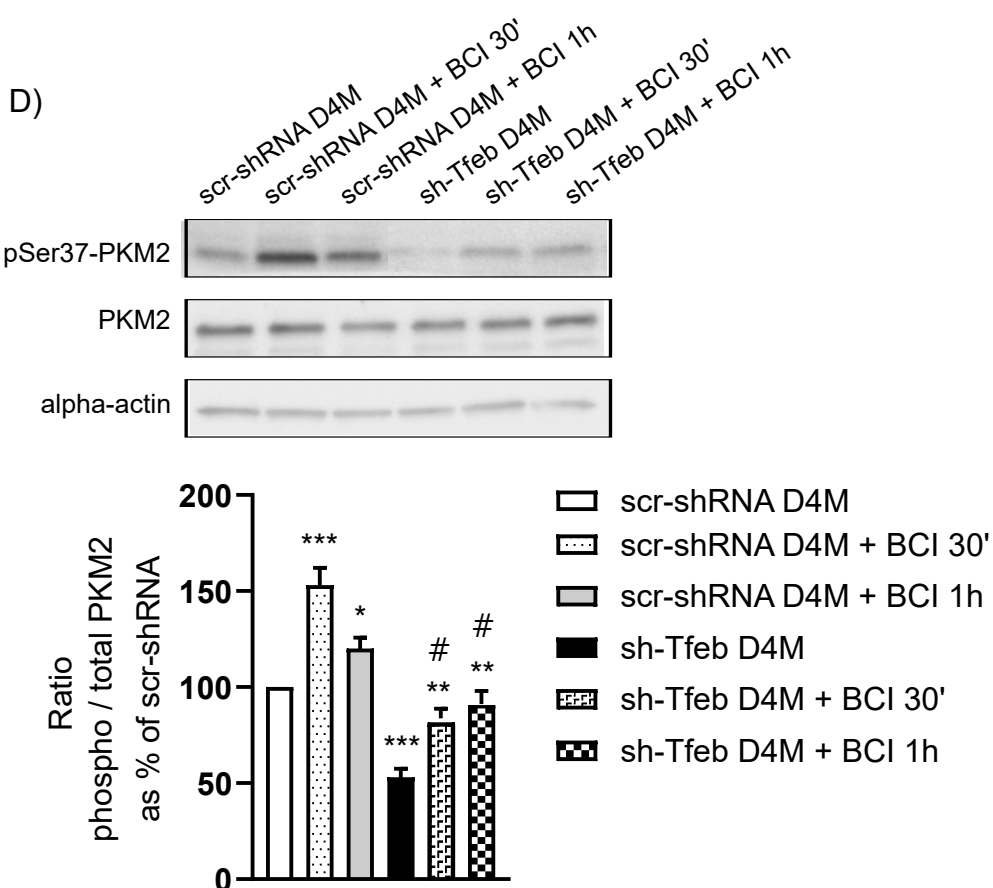

Supplement: Supplementary file 5 — SUPPLEMENTAL FIG S4 [file 41419_2023_5828_MOESM5_ESM.pdf]

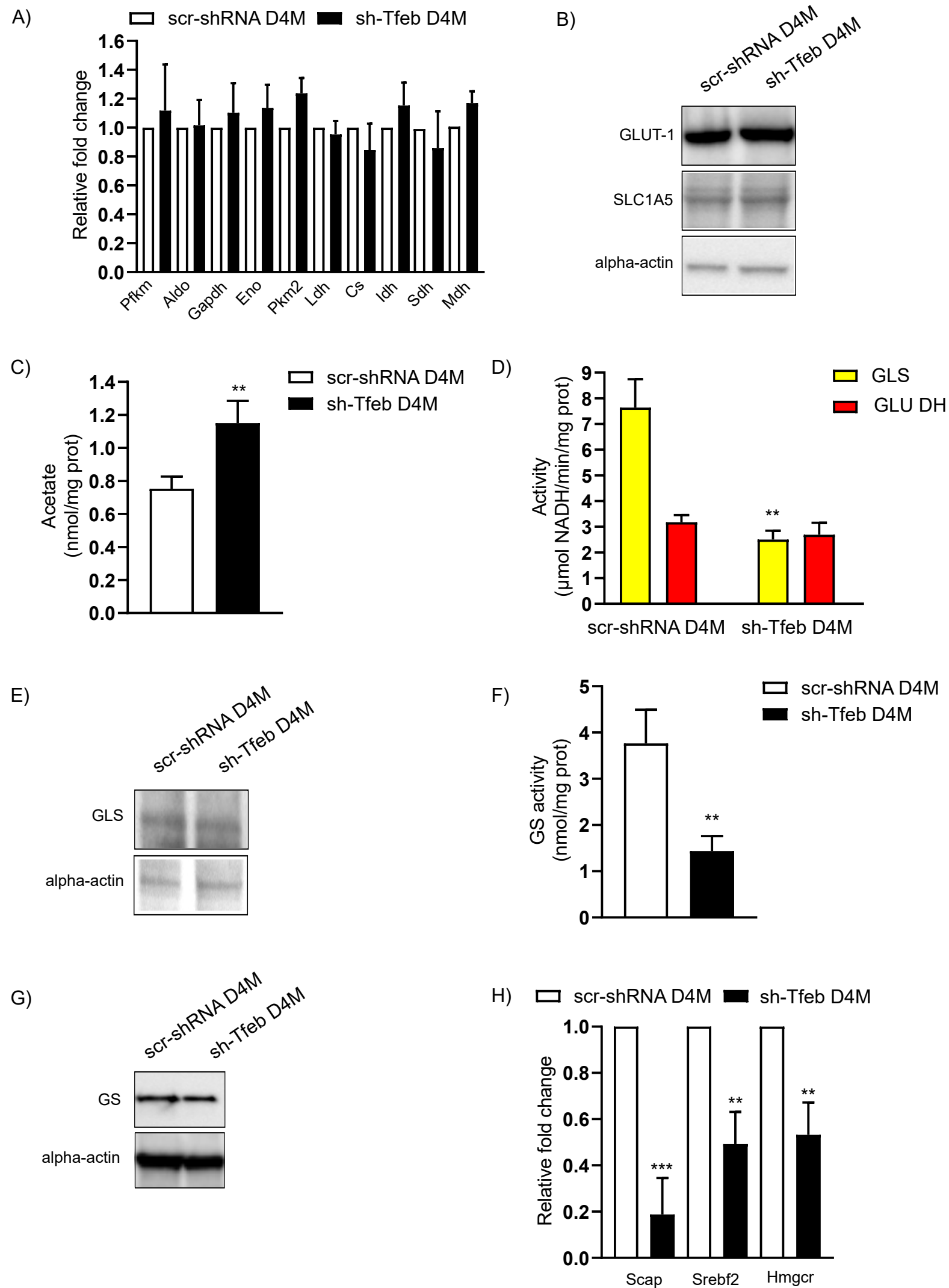

FIG. S5

Supplement: Supplementary file 6 — SUPPLEMENTAL FIG S5 [file 41419_2023_5828_MOESM6_ESM.pdf]

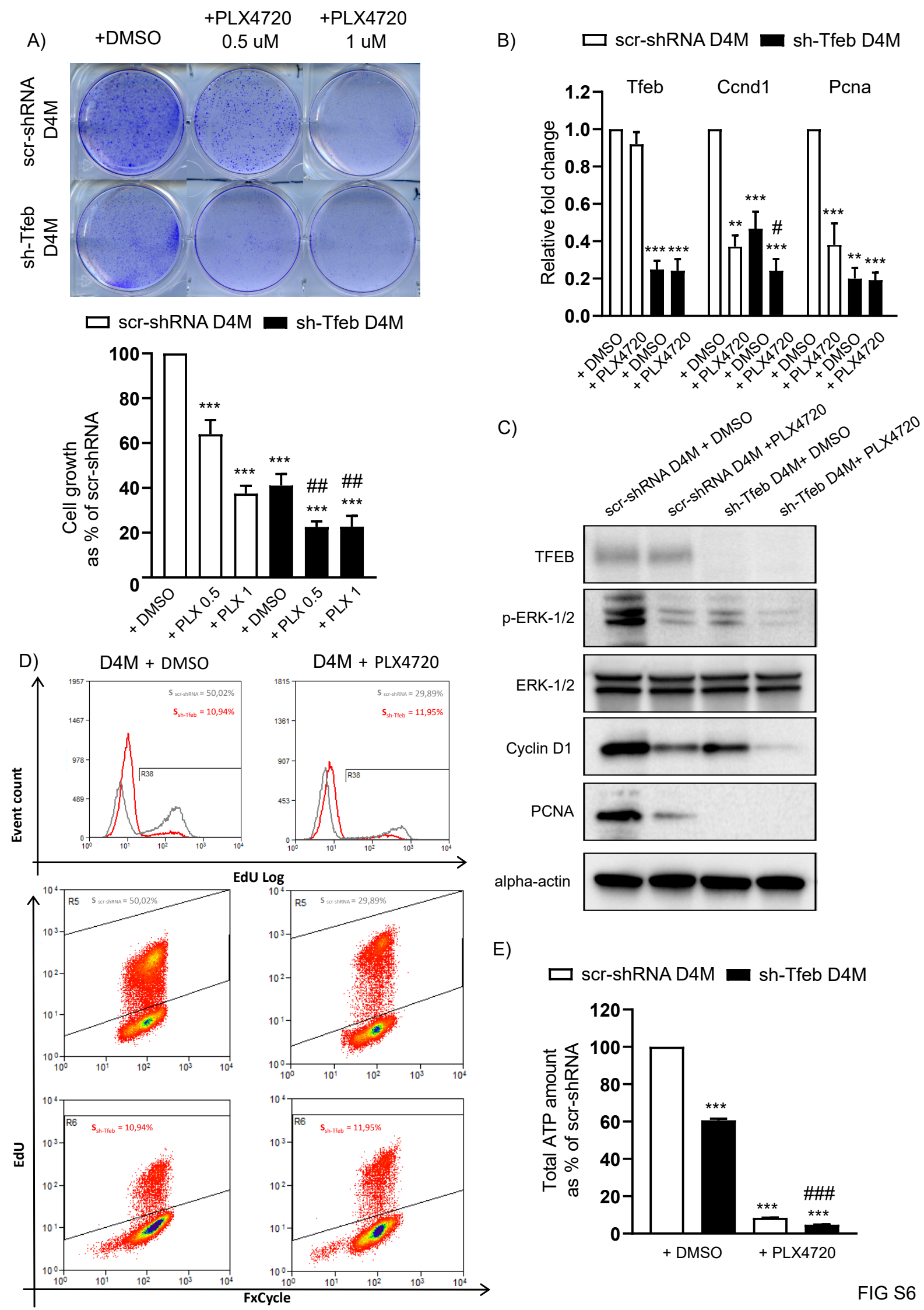

Supplement: Supplementary file 7 — SUPPLEMENTAL FIG S6 [file 41419_2023_5828_MOESM7_ESM.pdf]

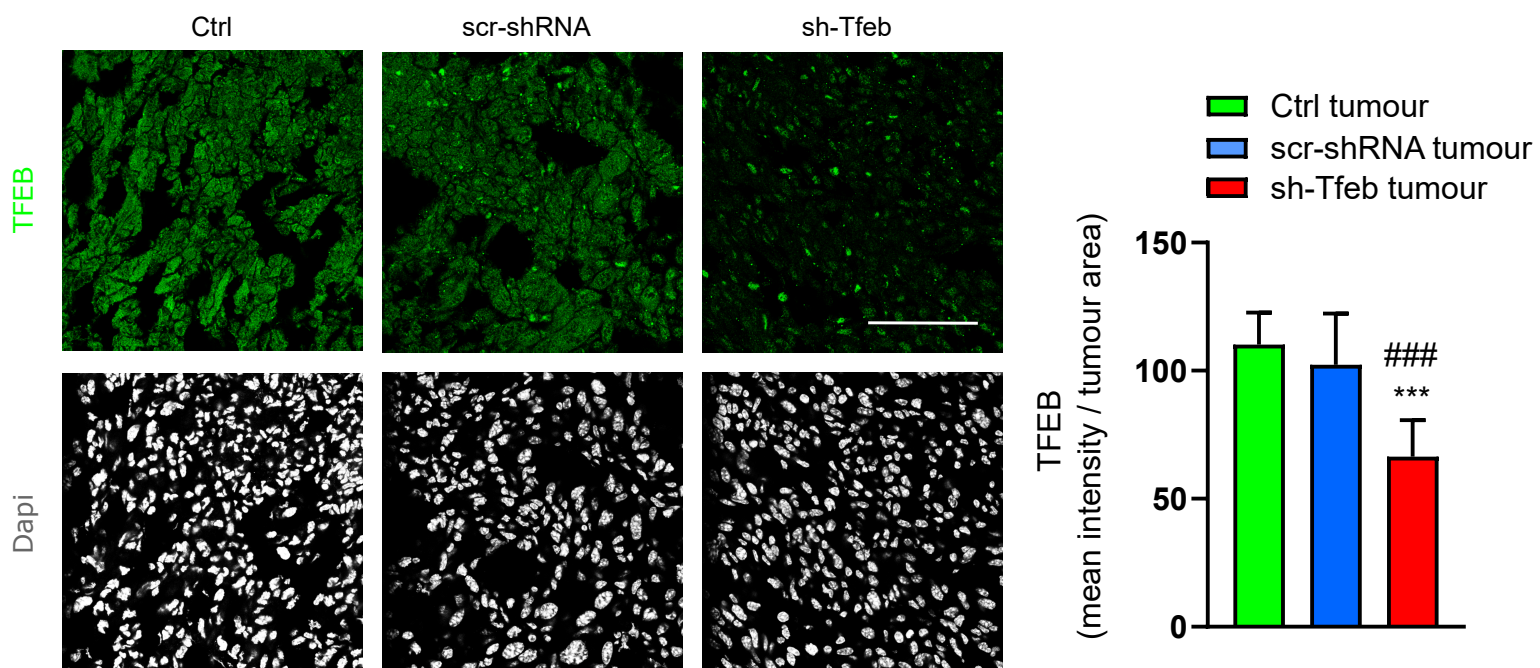

Supplement: Supplementary file 8 — SUPPLEMENTAL FIG S7 [file 41419_2023_5828_MOESM8_ESM.pdf]

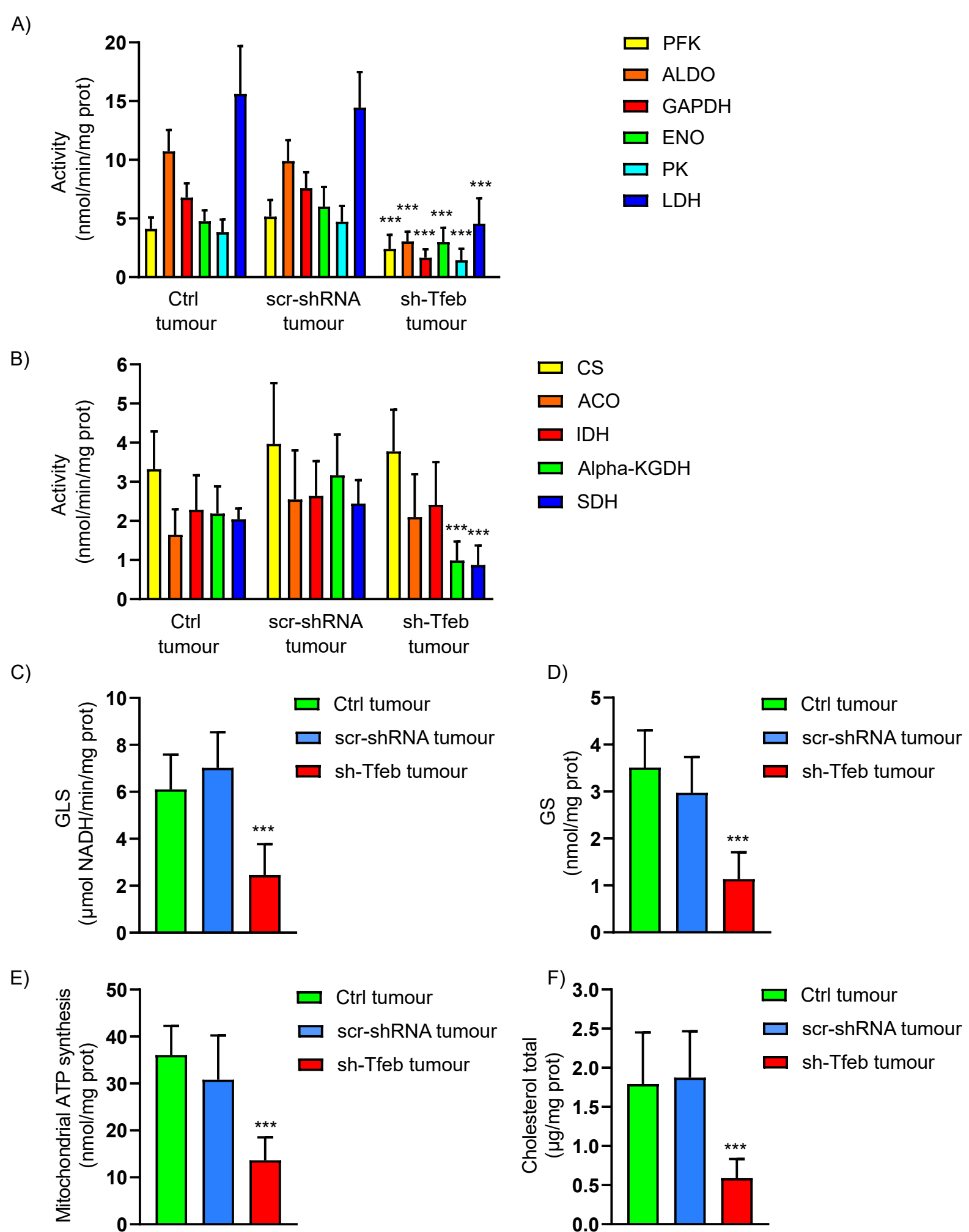

FIG. S8

Supplement: Supplementary file 9 — SUPPLEMENTAL FIG S8 [file 41419_2023_5828_MOESM9_ESM.pdf]

Fig 1A

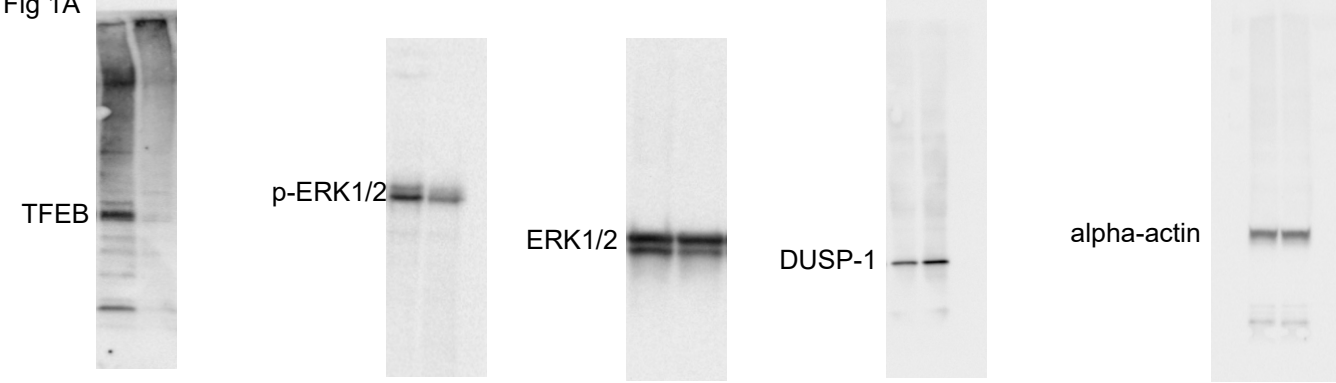

Fig 1D

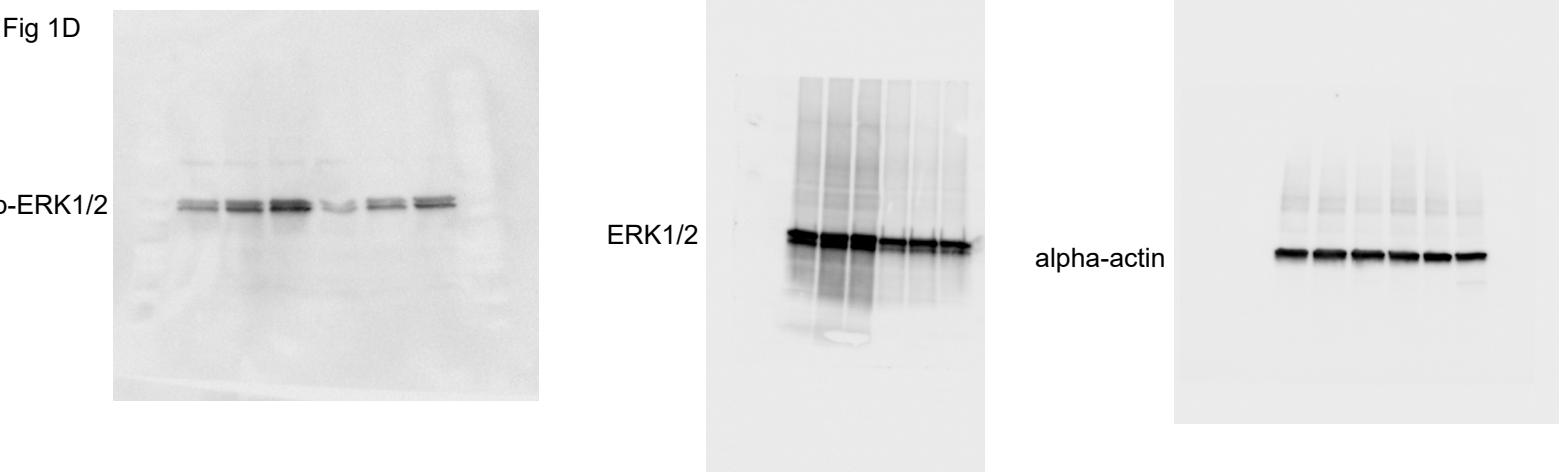

Fig 2C

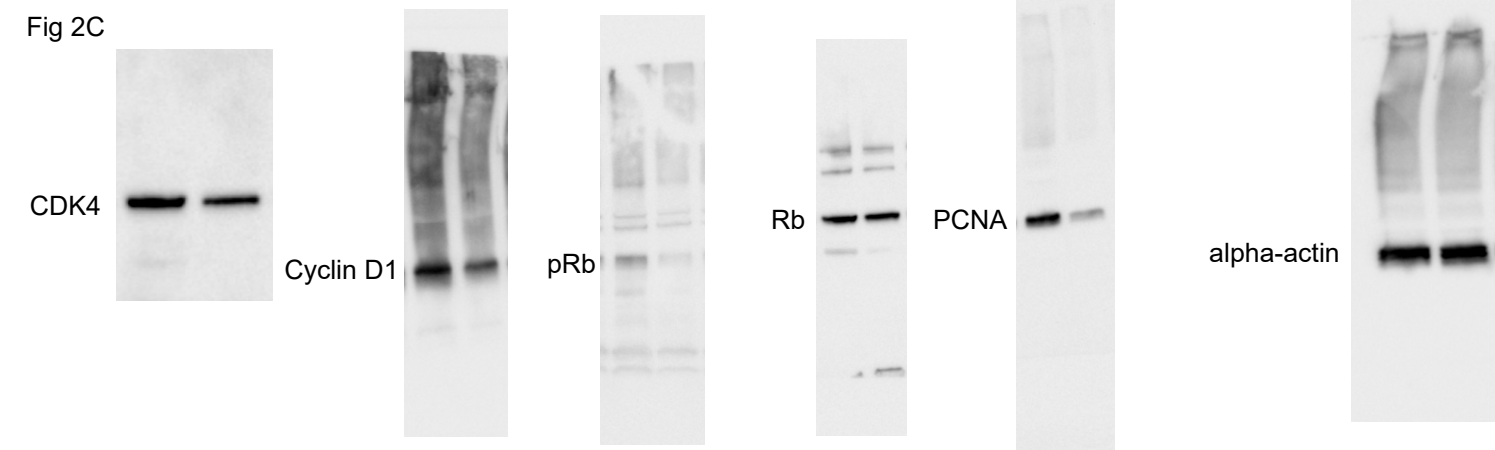

Fig 2E

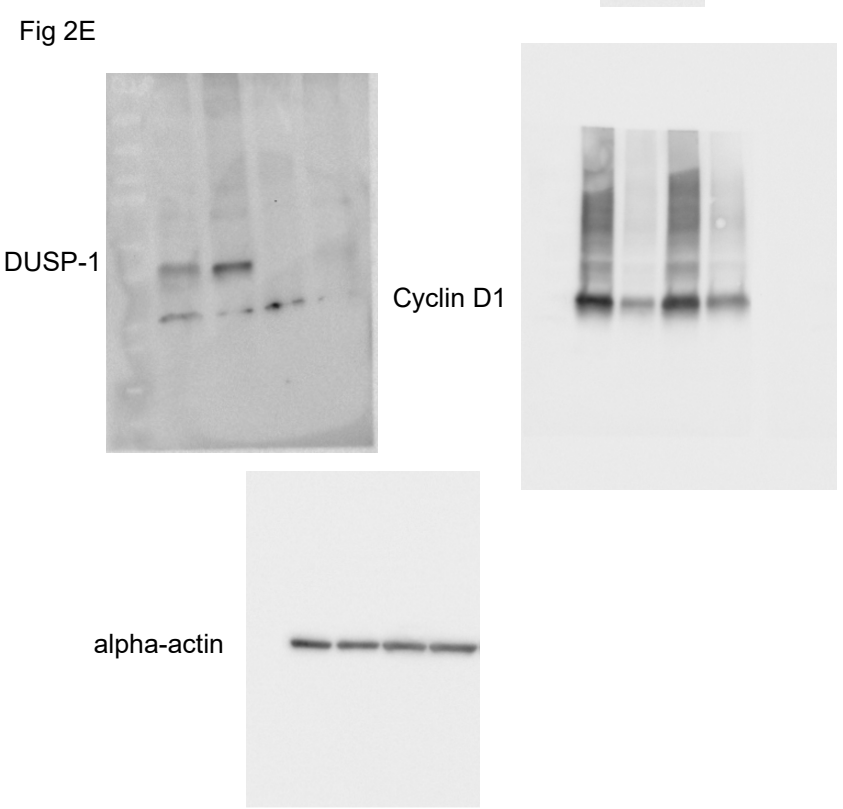

Fig 6A

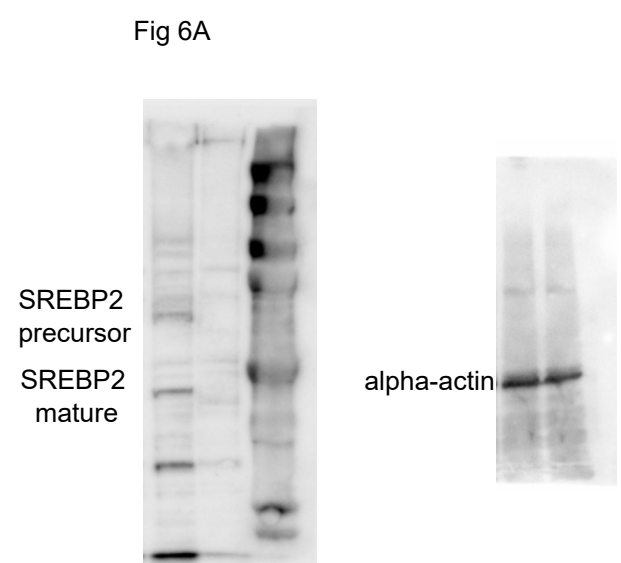

Fig S1A

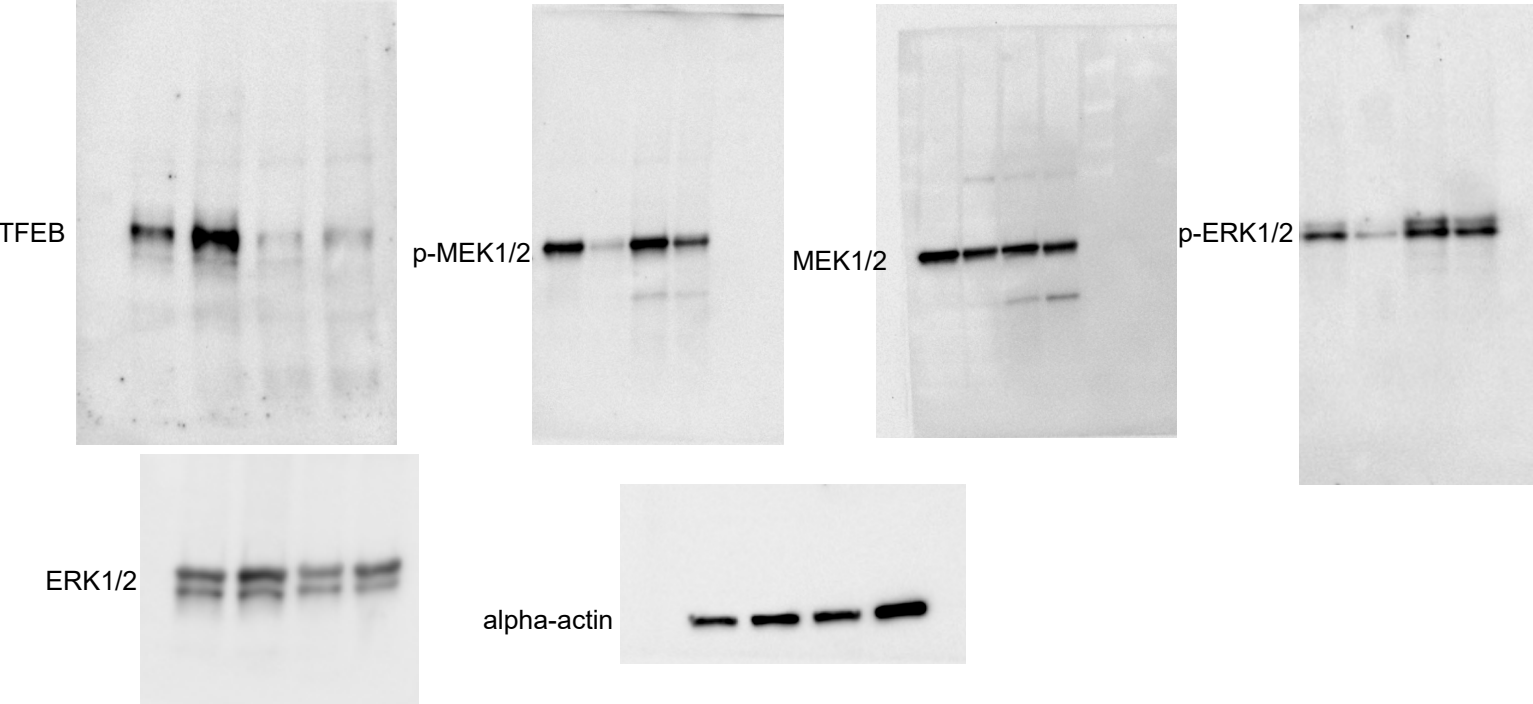

Fig S1D

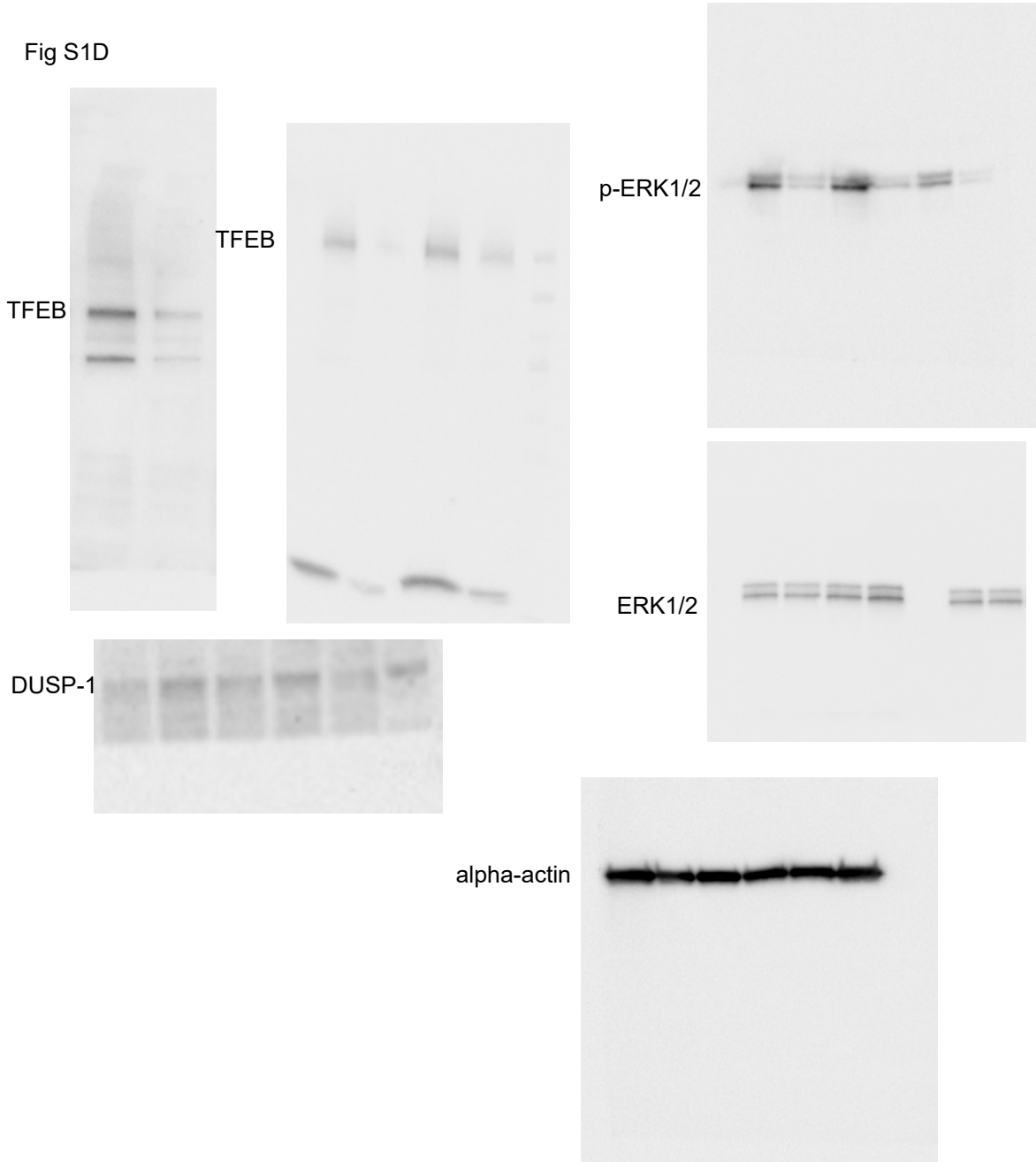

Fig S1E

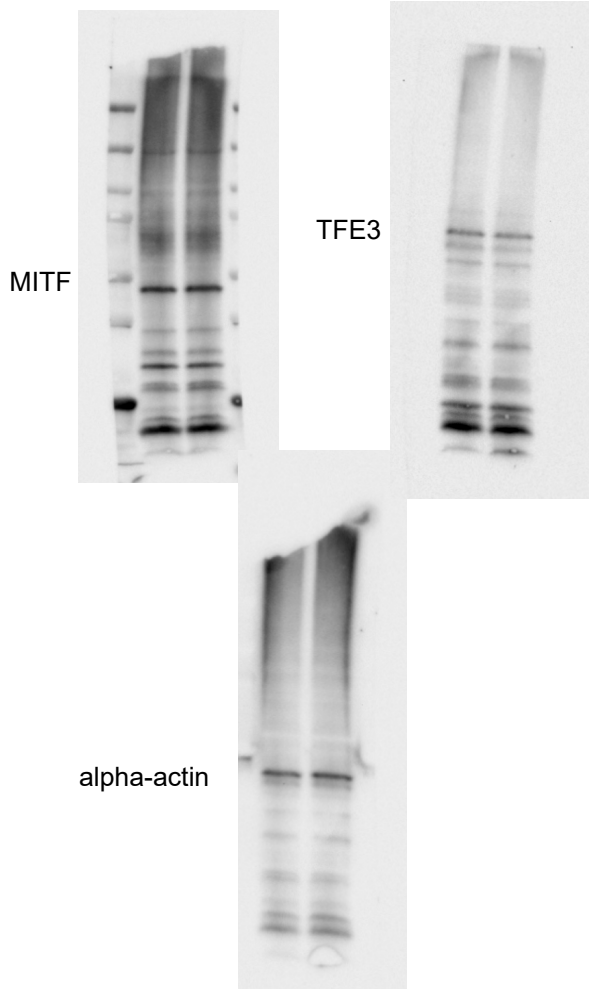

Fig S4D

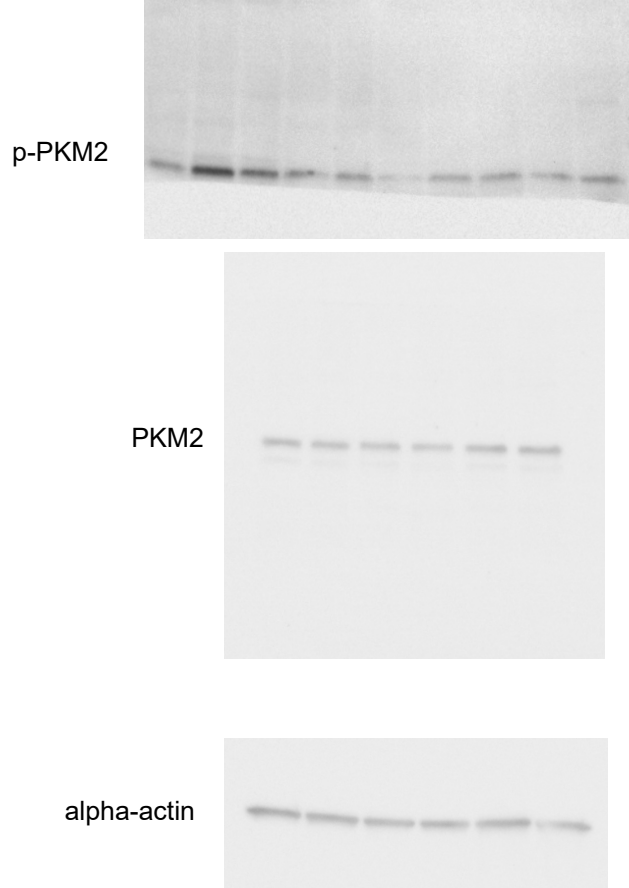

Fig S5B

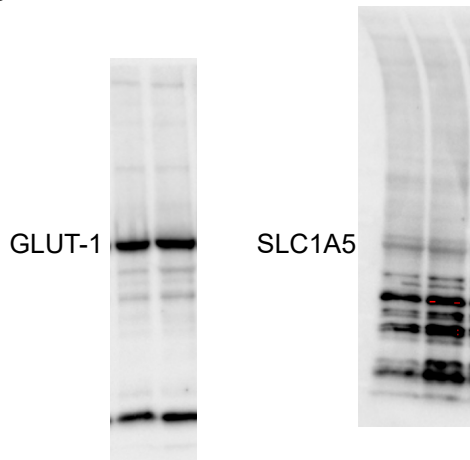

Fig S5E

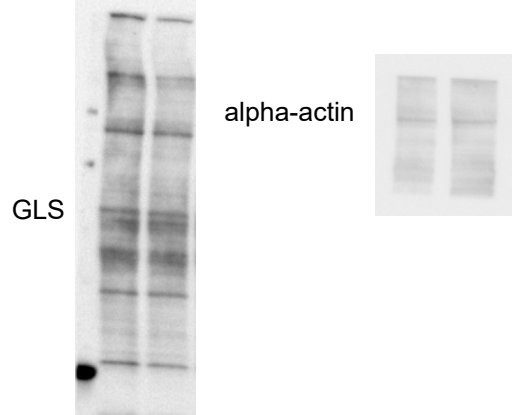

Fig S5F

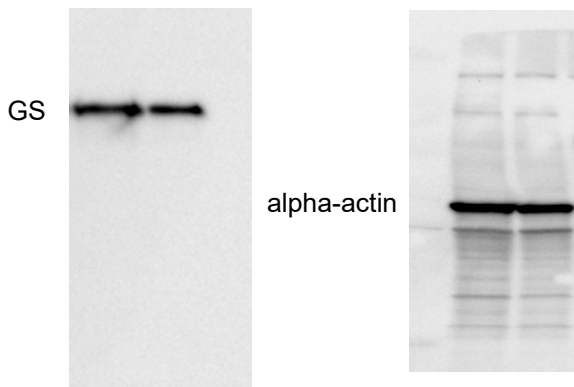

Fig S6C

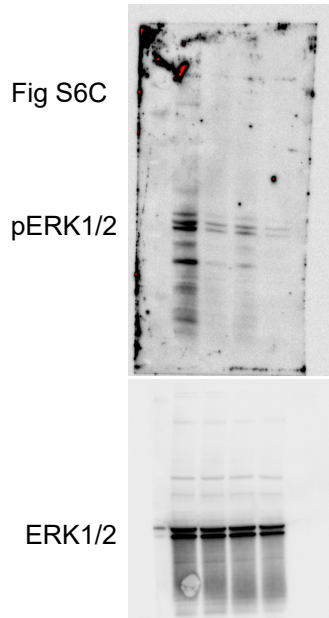

TFEB

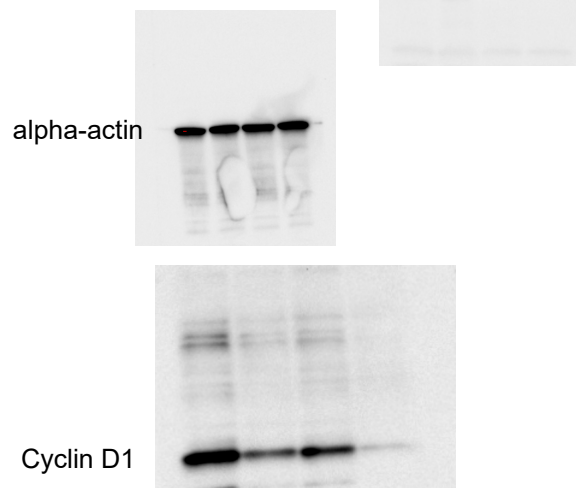

Supplement: Supplementary file 10 — FIG WESTERN BLOT [file 41419_2023_5828_MOESM10_ESM.pdf]
