# Supplementary material for: TFEB inhibition induces melanoma shut-down by blocking the cell cycle and rewiring metabolism
Source: Cell Death Dis. 2023 May 9;14(5):314. doi: 10.1038/s41419-023-05828-7 (PMC10170071; doi:10.1038/s41419-023-05828-7)
Supplement: Supplementary file 3 — SUPPLEMENTAL FIG S2 [file 41419_2023_5828_MOESM3_ESM.pdf]

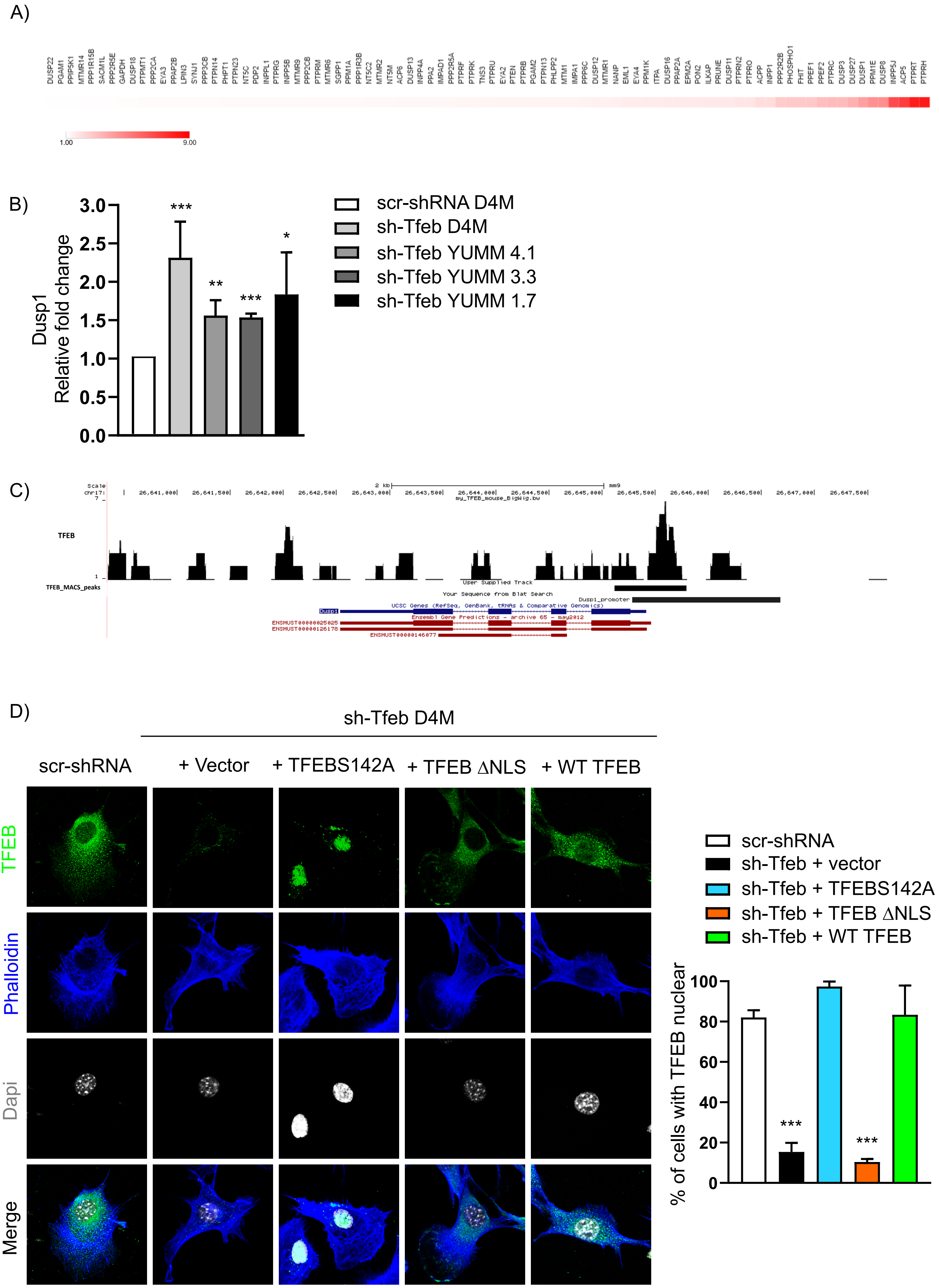
A)
DUSP22
PGAM1
PPIP5K1
MTMR14
PPP1R15B
SACM1L
PPP2R5E
GAPDH
DUSP18
PTPMT1
PPP2CA
EYA3
PPAP2B
LPIN3
SYNJ1
PPP3CB
PTPN14
PHPT1
PTPN23
NT5C
PDP2
INPPL1
PTPRG
INPP5B
MTMR9
PPP2CB
PTPRM
MTMR6
SGPP1
PPM1A
PPP1R3B
NT5C2
MTMR2
NT5M
ACP6
DUSP13
INPP4A
PPA2
IMPAD1
PPP2R5A
PTPRF
PTPRK
TNS3
PTPRU
EYA2
PTEN
PTPRB
PGAM2
PTPN13
PHLPP2
MTM1
IMPA1
PPP6C
DUSP12
MTMR1
NANP
EML1
EYA4
PPM1K
ITPA
DUSP16
PPAP2A
EPM2A
PON2
ILKAP
PRUNE
DUSP11
PTPRN2
PTPRO
ACPP
INPP1
PPP2R2B
PHOSPHO1
FHIT
PPEF1
PPEF2
PTPRC
DUSP3
DUSP27
DUSP1
PPM1E
DUSP8
INPP5J
ACP5
PTPRT
PTPRH
1.00
9.00
B)
Dusp1
Relative fold change
3.0
2.5
2.0
1.5
1.0
0.5
0.0
***
**
***
*
scr-shRNA D4M
sh-Tfeb D4M
sh-Tfeb YUMM 4.1
sh-Tfeb YUMM 3.3
sh-Tfeb YUMM 1.7
C)
TFEB
TFEB_MACS_peaks
User Supplied Track
Your Sequence from Blat Search
Dusp1_promoter
UCSC Genes (RefSeq, GenBank, tRNAs & Comparative Genomics)
Dusp1
Ensembl Gene Predictions - archive 65 - may2012
ENSMUST00000025025
ENSMUST00000126178
ENSMUST00000146077
D)
sh-Tfeb D4M
scr-shRNA
+ Vector
+ TFEBS142A
+ TFEB ΔNLS
+ WT TFEB
TFEB
Phalloidin
Dapi
Merge
scr-shRNA
sh-Tfeb + vector
sh-Tfeb + TFEBS142A
sh-Tfeb + TFEB ΔNLS
sh-Tfeb + WT TFEB
% of cells with TFEB nuclear
100
80
60
40
20
0
***
***

FIG. S2
